# Metagenomic analysis of water column samples collected from Green Canyon 233 prior to the Deepwater Horizon incident

**DOI:** 10.1128/aem.00799-26

**Published:** 2026-06-18

**Authors:** Shen Jean Lim, Luke R. Thompson, Kelly Goodwin

**Affiliations:** 1 Ocean Chemistry and Ecosystems Division, Atlantic Oceanographic and Meteorological Laboratory, National Oceanic and Atmospheric Administration, Miami, Florida, USA; 2 Cooperative Institute for Marine and Atmospheric Studies, Rosenstiel School of Marine, Atmospheric, and Earth Science, University of Miami, Miami, Florida, USA; 3 Northern Gulf Institute, Mississippi State University 5547, Mississippi State, Mississippi, USA; 4 NOAA Ocean Exploration, National Oceanic and Atmospheric Administration, La Jolla, California, USA; University of Delaware, Lewes, Delaware, USA

**Keywords:** hydrocarbon degradation, metagenomics, MAG, Deepwater Horizon, oil spill, Gulf of Mexico, Gulf of America, baseline

## Abstract

The Gulf of Mexico/Gulf of America provides ecosystem services derived from marine biodiversity and oil and gas resources. Threats posed by unintended releases of oil and gas can be attenuated by microbial processes, necessitating the documentation of baseline microbial diversity to better understand spill dynamics and to inform bioremediation strategies. Here, we analyze metagenomic sequencing of 10 water column samples collected from the Green Canyon 233 (GC233) lease block near the mussel-fringed brine lake, Brine Pool NR-1. Bioinformatics processing produced 60 bacterial metagenome-assembled genomes (MAGs), 11 archaeal MAGs, 149 microbial taxa predicted from assembled full-length small subunit (SSU) rRNA genes, and 389 microbial genera predicted from single-copy marker genes. Abundant taxa classified from these analyses included archaeal Nitrosopumilaceae, *Nitrosopelagicus,* and Thalassarchaeaceae and the bacterial taxa Pelagibacteraceae and SAR324. The MAGs revealed genes that degrade gaseous and non-gaseous hydrocarbons, including methane, other alkanes, and aromatic compounds. These samples were collected in 2009, fortuitously prior to the 2010 Deepwater Horizon (DWH) oil spill. Therefore, we searched for members of the rare biosphere that dominated the DWH plume during the early phase of microbial succession. Sequences related to *Bermanella* spp. were not detected initially. The search was expanded by mapping reads from ours and an additional 55 metagenomic libraries to two *Bermanella* MAGs. Read recruitment to *Bermanella sp913054445* enriched in DWH plume samples was low (<1%) for our samples, those collected after the spill, and most experimental samples compared to samples collected outside (3%) and inside the DWH plume (19%–23%) during the spill.

**IMPORTANCE:**

Microbes execute oil spill biodegradation through complex interactions involving whole microbiome communities by harnessing genes distributed across multiple taxa. Therefore, metagenomic data sets provide taxonomic and functional annotations to aid in understanding spill dynamics. Although the Deepwater Horizon oil spill provided opportunities to observe ecosystem recovery, data about the microbiome prior to the spill are scarce and limited to amplicon sequencing. Our metagenomic libraries, although not derived from the same lease block as the blowout, contribute linkages between microbial taxonomy and function in an area of active oil and gas production. This analysis can aid microbial indicator development, machine learning, and modeling efforts to bioremediate hydrocarbon influxes in marine environments.

## INTRODUCTION

Microbiomes can attenuate accidental releases of oil and gas, driving a need to understand microbial diversity to inform remediation strategies (1, 2). Evaluating mitigation strategies requires systematic comparisons of microbial communities and their functional potential before, during, and after a spill. Baseline knowledge is important for assessing ecosystem changes, identifying past and present stressors, predicting environmental impacts, and guiding restoration goals (3).

The Gulf of Mexico/Gulf of America is one of the most ecologically and economically important marine ecosystems in the world. The Gulf continental slope contains rich oil and gas reserves (4), which support numerous natural seeps (5, 6) and fuel a thriving petroleum industry (7). Natural seeps in the Gulf support rich chemosynthetic ecosystems that have been a focus of study since first described in the mid-1980s (8, 9). Here, we provide taxonomic and functional profiles through metagenomic analysis of single-copy marker genes, assembled full-length 16S rRNA gene sequences, and metagenome-assembled genomes (MAGs) from libraries generated from water column samples collected from the Green Canyon area of the Gulf, including samples collected over Brine Pool NR-1. This hypoxic brine lake contains high concentrations of methane (8) and is lined with the mussel *Bathymodiolus childressi* harboring methanotrophic gill symbionts (8, 10). Microbial communities capable of sulfate reduction, acetogenesis, and methanogenesis have been identified in the brine (11).

Although natural seeps are the main source of petroleum hydrocarbons in the ocean, anthropogenic activities associated with oil and gas extraction, transport, and consumption can cause pollution with detrimental impacts on marine biodiversity (7). However, a lack of baseline data of pre-spill conditions makes it difficult to assess ecological impacts. To date, the two largest oil spills in the Gulf were the Ixtoc-I spill in 1979–1980 and the Deepwater Horizon (DWH) spill in 2010. The Ixtoc-I spill flowed for approximately 290 days, releasing more than 3.4 million barrels (~540 million L) of crude oil (12). The DWH spill (13) released approximately 795 million liters of oil, resulting in at least 1,046 km of oiled coastline (14), with 2.9 million L of chemical dispersant additionally applied (1, 14–16). It is predicted that rising demand for global energy and regional economic growth will continue to increase pollution risks to the Gulf, including from oil spills (2, 17). Metagenomic data can improve insight into microbial community taxonomy and function, which uses cooperative metabolism to execute degradative pathways (3). In turn, this understanding can inform intervention approaches designed to accelerate bioremediation (3).

Prior to the DWH spill, the availability of bacterial sequence data appears limited to amplicon sequencing. For example, a 16S rRNA gene clone library was obtained from a single water sample serendipitously collected in March 2010, approximately 1 month before the spill, at 800 m depth, and 9–10 nautical miles northwest of the Macondo wellhead (18). A similar microbial composition was reported for this sample and the Atlantic Ocean, the source reservoir for the Gulf (18). Dominant sequences from these clones included Pelagibacterales (formerly SAR11) and other Alphaproteobacteria, phylum Fidelibacterota (formerly SAR406), and SAR324 (formerly Deltaproteobacteria) (19), and cultured and uncultured gammaproteobacterial taxa such as Alteromonadales and Oceanospirillales, now merged into order Pseudomonadales (20). Other phyla sequenced included Acidobacteriota, Bacteroidota, Chloroflexota, Cyanobacteriota, Gemmatimonadota, Planctomycetota, and Verrucomicrobiota (18) (published under former taxonomies).

In another pre-DWH study, the 16S rRNA V4 gene region was amplified from water samples collected in March 2010 from 17 stations in the northern Gulf of Mexico (21, 22). This analysis revealed distinct microbial compositions in samples collected from ≤100 m depth compared to those collected from deeper waters (21, 22). Shallow water samples were dominated by Bacteroidota and Pelagibacterales, while Nitrososphaeria, Bacillota, and Gammaproteobacteria were more abundant in deeper water samples. However, significant differences by location or depth were not observed with amplification of alkane hydroxylase (*alkB*) (23). Although *alkB* sequences similar to those found in hydrocarbon-degrading *Alcanivorax borkumensis* and *Marinobacter* dominated that data set, these sequences were not detected in plume metagenomes (24) or metatranscriptomes (25) associated with the spill (21, 22). Hydrocarbon-degrading genera such as *Cycloclasticus, Methylobacter*, *Methylococcus*, and a Pseudomonadota taxon related to *Oleispira* were identified in pre-spill water samples (21, 22), as well as 16S rRNA gene amplicons (26, 27), metagenomic sequences (24), and metatranscriptomic libraries (25) from DWH plume samples.

Our samples were fortuitously collected in 2009 prior to the DWH oil spill; therefore, we employed metagenomic analysis to glimpse the microbial community in these historical samples. In particular, we searched for sequences related to uncultured Oceanospirillales/Pseudomonadales and *Bermanella* spp., which were identified as early responders to the spill. These taxa showed enrichment in the DWH plume (24, 28), after the well was capped (29, 30), and during experiments (31–33) designed to recapitulate spill conditions. Although collected from a different lease block as the DWH blowout, these data provide functional and taxonomic insights into historical Gulf samples collected in an area with active oil and gas production.

## MATERIALS AND METHODS

### Sample collection

**Fig 1:**
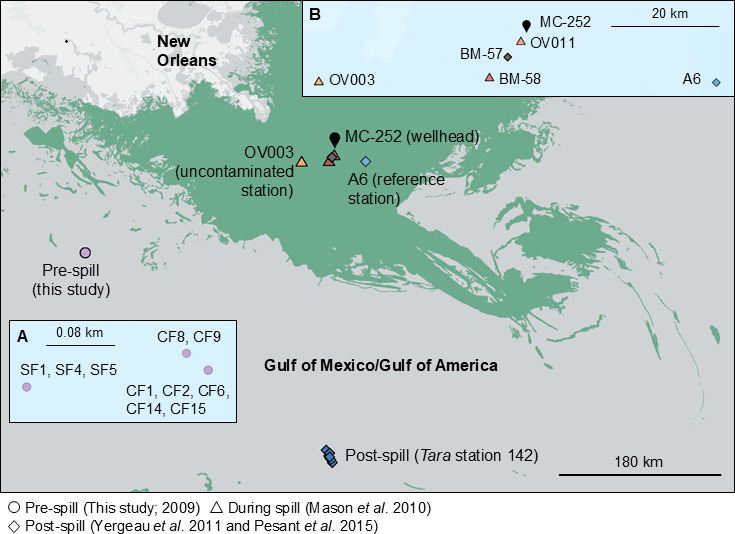
Map showing where the samples were collected for this study (circle markers; inset A) and other sites where the samples were collected during (triangle markers) and after (diamond markers) the DWH oil spill (inset B), which originated at the Mississippi canyon MC-252 wellhead (black marker). Libraries sequenced from samples collected from our study were used for taxonomic and diversity analyses, while libraries sequenced from samples collected from other sites were used for read recruitment analyses to investigate the environmental reservoir of *Bermanella* spp. The green area shows the potential oiling footprint of the DWH oil spill from April to August 2010, as captured by National Environmental Satellite Data and Information Service synthetic aperture radar (NESDIS SAR). Map sources: Esri, CGIAR, TomTom, Garmin, FAO, NOAA, USGS, ©OpenStreetMap contributors, and the GIS User Community.

Epipelagic (198–217 m) and mesopelagic (640–655 m) samples were collected by Niskin from two separate conductivity, temperature, and depth (CTD) casts aboard the R/V *Seward Johnson*. One cast collected samples (SF1, SF4, and SF5) above Brine Pool NR-1, a cold seep located on the northern Gulf Upper Continental Slope (8). In addition, a Niskin mounted on the remotely operated underwater vehicle (ROV), the *Johnson Sea Link*, was employed to collect samples ~3 m outside the brine pool (CF8 and CF9) (Table 1; Table S1). All samples were collected in 2009 from the Gulf of Mexico Green Canyon 233 Mineral Management Service lease block (GC233), which is >100 miles southwest of the Macondo wellhead and outside the identified DWH plume (Fig. 1).

**TABLE 1:** Description and environmental data associated with the samples collected for this study^*a*^

Sample	ID	Description	Latitude	Longitude	Collection time (UTC)	Depth (m)	Temperature (°C)	Fluorescence (mg/m^3^)	Irradiance (PAR)	Oxygen (ml/L)	Salinity (psu)
SF1	SJ09 CTD4-01-19	CTD4 bottle 19, ~430 m above Brine Pool NR-1	27.72335	−91.28117	19:07:47	217	15.3722	3.85E-02	1.78E-02	2.73461	36.0081
SF5	SJ09 CTD4-01-3	CTD4 bottle 3, ~10 m above Brine Pool NR-1	27.72335	−91.28117	18:24:43	640	7.3460	4.72E-02	0.00E + 00	2.57682	34.9306
SF4	SJ09 CTD4-01-1	CTD4 bottle 1, at Brine Pool NR-1	27.72335	−91.28117	18:22:28	645	7.3309	4.69E-02	0.00E + 00	2.58603	34.9303
CF8, CF9	DIVE 3752	ROV Dive 3752 Niskin ~ 3 m west of outer edge of Brine Pool NR-1 mussel bed	27.72371	−91.27926	17:53	649	n/a	n/a	n/a	n/a	n/a
CF1, CF2	SJ09 CTD8-01-11	CTD8, bottle 11	27.72353	−91.279	19:21:31	198	16.6481	4.34E-02	9.99E-02	2.80149	36.2069
CF6	SJ09 CTD8-01-2	CTD8, bottle 2	27.72353	−91.279	18:58:30	643	7.3959	5.20E-02	0.00E + 00	2.53473	34.9326
CF14, CF15	SJ09 CTD8-01-1	CTD8, bottle 1	27.72353	−91.279	18:56:57	652	7.1749	5.43E-02	0.00E + 00	2.57788	34.9262

^*a*^ Samples were collected in the GC233 area on September 29, 2009 (CTD4 and Dive 3752) or October 3, 2009 (CTD8). The volume of seawater filtered was 1 L, except for CF6, which was 500 mL. See Table S1 for additional information. PSU, practical salinity unit; ROV, remotely operated vehicle; PAR, photosynthetically active radiation; n/a, not available.

### DNA extraction and sequencing

Seawater was filtered on board through sterile 0.2-µm filters and stored at –80°C until DNA was extracted on Feb 8, 2012, using the method described in a study by Bostrom et al. (34). The 10 metagenomic libraries were sequenced on April 20, 2012, on two Illumina HiSeq 2000 lanes using 2 × 100 bp paired-end reads (Table S1).

### Read processing

Metagenomic reads from this study were quality-filtered and trimmed using Atropos v1.1.21 (35). The first base of the reverse read was trimmed from the 5′ end due to low quality in the first position. Next, low-quality (less than Q20) and ambiguous bases (Ns) were trimmed from 3′ ends of both reads, and only sequences 50 bp or greater were retained. Read quality was checked before and after quality filtering using FastQC v0.11.8 (https://www.bioinformatics.babraham.ac.uk/projects/fastqc/) and MultiQC v1.7 (36).

### *k*-mer profiling

Simka v1.5.1 (37) was used to compute *k*-mer counts from quality-trimmed reads and calculate the ecological distances between samples based on *k*-mer profiles. The pre-computed Bray-Curtis dissimilarity matrix generated by Simka was imported into R v4.5.1 as a distance matrix, and the principal coordinate decomposition of the Bray-Curtis dissimilarity matrix was calculated using the pcoa function in the APE v5.8.1 (38) R package prior to ordination analysis using the phyloseq v1.52.0 (39) R package, as described in “Ordination analyses,” below.

### Read-based taxonomic composition profiling

Taxonomic composition was analyzed using SingleM v0.20.3 (40). As recommended by the user manual, raw untrimmed reads were used because quality-trimmed reads are often too short for operational taxonomic unit (OTU) assignment. The SingleM pipe command was used to assign OTUs to raw untrimmed reads for each sample by identifying conserved sections corresponding to 35 bacterial and 37 archaeal single-copy marker genes, assigning taxonomy to each OTU using the Genome Taxonomy Database (GTDB) release 226 (41) reference database, and converting marker-specific OTU tables into a condensed taxonomic profile. The SingleM summarize command was used to convert the condensed taxonomic profile into a relative abundance table that was collapsed at various taxonomic levels (Data Set S1). Ordination was performed as described in “Ordination analyses,” below.

### Alpha diversity profiling

To measure alpha diversity, the SingleM genus relative abundance, taxonomic, and metadata tables were imported and converted into a phyloseq (39) object. Alpha diversity indices, including observed OTUs, Shannon index (42), and the Inverse Simpson index (43) were computed using the phyloseq (39) plot_richness command. The distribution of each alpha diversity index was tested for normality using the Shapiro-Wilk test (44), which did not reject the null hypothesis that each data set was normally distributed. Therefore, the parametric Student’s two-sample *t*-test (45) was used to determine whether observed OTU and Shannon index values were statistically significant between the epipelagic and mesopelagic samples.

### Small subunit (SSU) rRNA sequence assembly

Raw untrimmed reads were assembled into full-length bacterial/archaeal/eukaryotic SSU rRNA sequences using the default parameters of SPAdes implemented in phyloFlash v3.4 (46). Assembled SSU sequences classified under the class Gammaproteobacteria were compared with three 16S rRNA gene sequences (HM587888, HM587889, and HM587890) of uncultured Pseudomonadales bacterium clones sequenced from proximal and distal stations during the spill (26) using local blastn searches implemented in the NCBI BLAST +v2.11.0+ (47).

### Metagenomic assembly and binning

Quality-trimmed reads for our 10 libraries (Table 1) were co-assembled by MEGAHIT v1.2.9 (48) using the meta-large preset option for large and complex communities, and quality was assessed using QUAST v5.0.2 (49). Each co-assembled metagenome was binned with the default parameters of MaxBin v2.2.7 (50) and MetaBat v2.12.1 (51) using contig length thresholds of 1,500, 2,000, and 2,500 bp. Prior to binning with MetaBat2, quality-trimmed reads were mapped to contigs ≥1,000 bp in each assembled metagenome using Bowtie2 v2.4.2 (52) and SAMtools v1.12 (53). MAGs generated by MaxBin2 and MetaBat2 at different contig length thresholds were de-replicated and optimized using the default parameters of DASTool v1.1.2 (54). MAG quality was evaluated with CheckM v1.1.3 (55), and the taxonomy of each MAG was predicted using the Genome Taxonomy Database toolkit (GTDB-Tk v2.4.0) (56) based on GTDB release 220.

### Read recruitment of gammaproteobacterial genomes

Competitive recruitment employed the sequences from this study (Table 1) and an additional 55 libraries sequenced from samples collected during or after the DWH spill or during incubation and simulation experiments (Table S1). Reads were mapped against MAGs assembled from the original Mason et al. data set (24), which contained 16S rRNA gene sequences matching clones isolated from the DWH plume (18) and assigned to *Bermanella sp913054445* by GTDB (28), including DWH *Oceanospirillales desum* (57), DWH *Bermanella* MAG BM58_1, DWH *Bermanella* MAG OV003_3, and DWH *Bermanella* MAG OV011_4. The analysis also included a MAG assembled from a simulation experiment, *Candidatus* Bermanella macondoprimitus (32) assigned to *Bermanella sp002162985* by GTDB. In detail, the reads were sourced from (i) four paired-end libraries sequenced by Mason et al. (24) from a distal plume sample, a proximal plume sample, and two uncontaminated seawater samples collected at the depth of the DWH plume in May 2010; (ii) six paired-end libraries sequenced by Dombrowski et al. (31) from two plume samples incubated with ^13^C-labeled *n*-hexadecane, two surface seawater samples incubated with ^13^C-labeled naphthalene, and two surface seawater samples incubated with ^13^C-labeled phenanthrene collected during the spill in May 2010; (iii) three paired-end libraries sequenced by Hu et al. (32) from seawater collected from MC-294 at plume depth mixed with simulated microdroplets of Macondo oil and Corexit EC9500A dispersant at days 6, 18, and 64; (4) 30 single-end libraries sequenced by Yergeau et al. (30) from 15 seawater samples collected from station BM-57 3.78 km southwest of the wellhead and 15 seawater samples collected from reference station A6 located 37.77 km southeast of the wellhead at 1–2,174 m depth in September 2011, 1 year after the well was capped; and (v) 12 paired-end libraries sequenced by the *Tara* Oceans Consortium (29) from seawater samples collected from the Gulf of Mexico/Gulf of America (station 142) at 5–640 m depth during the *Tara* Oceans expedition in January 2012 after the well was capped (Fig. 1; Table S1).

All reads were trimmed as described in the “Read processing” subsection above. Metagenomic reads from the *Tara* Oceans expedition (29) were already quality-trimmed by the data owners and were not further processed in this analysis. Reads from each library were mapped to contigs in each *Bermanella* MAG using Bowtie2 v2.5.4 (52) and SAMtools v1.21 (53). The BAM files and MAG sequence files were imported into anvi'o v8 (57) using the anvi-profile, anvi-merge, anvi-import-collection, and anvi-summarize commands to calculate the percent recruitment of each genome in each library, which is the number of reads mapped to a genome divided by the total number of reads in a sample.

### Phylogenomic analysis of *Bermanella* genomes

Phylogenomic analysis was performed on 27 *Bermanella* genomes published in the literature or downloaded from the NCBI Genome database using the default parameters of Up-to-date Bacterial Core Genes (UBCG) v2, which includes a set of 81 bacterial core genes selected from 43 phyla (58). Core genes found in each genome set were automatically aligned, concatenated, and filtered to remove positions with >50% gaps. For each genome set, RAxML v8.2.12 (59) was invoked by UBCG2 to generate a phylogenomic tree (UBCG tree) using the default JTT + CAT model. Branch support was calculated using the Gene Support Index (GSI), which represents the number of single-gene trees supporting each branch in the final tree, with a maximum value of 81 corresponding to the number of bacterial core genes. The phylogenomic tree was visualized and annotated using FigTree v.1.4.4 (http://tree.bio.ed.ac.uk/software/figtree/).

### Metagenome/MAG annotation

HUMAnN 3.0 (60) was used to functionally annotate quality-trimmed reads from this study (Table 1) with MetaCyc (61) pathway abundances. Because HUMAnN 3.0 does not consider paired-end relationships, forward and reverse reads of paired-ends were concatenated and a joint taxonomic profile was constructed, as recommended (https://github.com/biobakery/humann). Briefly, MetaPhlAn (60) was used to create and merge taxonomic profiles to represent maximum abundances, and the HUMAnN 3.0 joint taxonomic profile with maximum abundances was run. The MetaCyc pathway abundance, taxonomic, and metadata tables generated from HUMAnN3 were used for ordination analysis.

The functional potential of each MAG from our samples (Table 1) was predicted using anvi'o (57), METABOLIC v4.0 (62), and the NCBI Prokaryotic Genome Annotation Pipeline (PGAP) (63). For anvi'o, the co-assembled metagenome was imported as a contigs database, together with alignment BAM files generated by mapping the trimmed reads to the contigs with Bowtie2 v2.4.2 (45) and SAMtools v1.12 (46), using the anvi-gen-contigs-database command. The anvi-run-ncbi-cogs command was used to assign NCBI’s Clusters of Orthologous Groups (COGs) functions to each open reading frame sequence predicted by Prodigal (64), based on local protein sequence alignments using DIAMOND (65). The anvi-run-kegg-kofams command was used to search open reading frame sequences predicted in each MAG against KOfam, a customized Hidden Markov Model (HMM) database of Kyoto Encyclopedia of Genes and Genomes (KEGG) Orthologs (KOs) (66). Subsequently, the anvi-estimate-metabolism command was used to predict KEGG module completeness in each MAG. KEGG pathway associations (66) for annotated genes were also provided automatically by METABOLIC (62). The KEGG modules and/or METABOLIC categories “aromatic degradation,” “C1 metabolism,” and “methane metabolism” were used to identify hydrocarbon degradation genes in the assembled MAGs. Potential hydrocarbon degradation genes were also identified by comparing gene names and sequences in assembled MAGs with those reported in the literature. Gene annotations by anvi'o (57), METABOLIC v4.0 (62), and the NCBI Prokaryotic Genome Annotation Pipeline (PGAP) (63) were checked for consistency and confirmed when functionally similar and taxonomically related homologs were identified using web BLAST searches (67) against NCBI’s nonredundant protein and nucleotide (nr/nt) databases (68). Gene clusters of interest were plotted in R v4.0.2 using the gggenes v0.4.1 (https://github.com/wilkox/gggenes) package. Methods for gene coverage analysis are described in the supplemental material.

### Ordination analyses

The Bray-Curtis dissimilarity (69), which accounts for OTU abundance differences between samples, was used in all ordination analyses performed in R v4.0.2 with the phyloseq v1.34.0 (39) package. For each phyloseq object, Bray-Curtis dissimilarities between samples were computed with lingoes correction using phyloseq’s ordinate function. Principal coordinates analysis (PCoA) plots were generated using phyloseq’s plot_ordination function. The non-parametric permutational multivariate analysis of variance (PERMANOVA) method implemented in the adonis2 function in the VEGAN v2.5.7 (70) R package was used to evaluate whether samples collected from 198 to 217 m depth and 640 to 655 m depth were significantly different from each other by partitioning the Bray-Curtis dissimilarity matrix among sources of variation.

## RESULTS

### Microbial diversity

**Fig 2:**
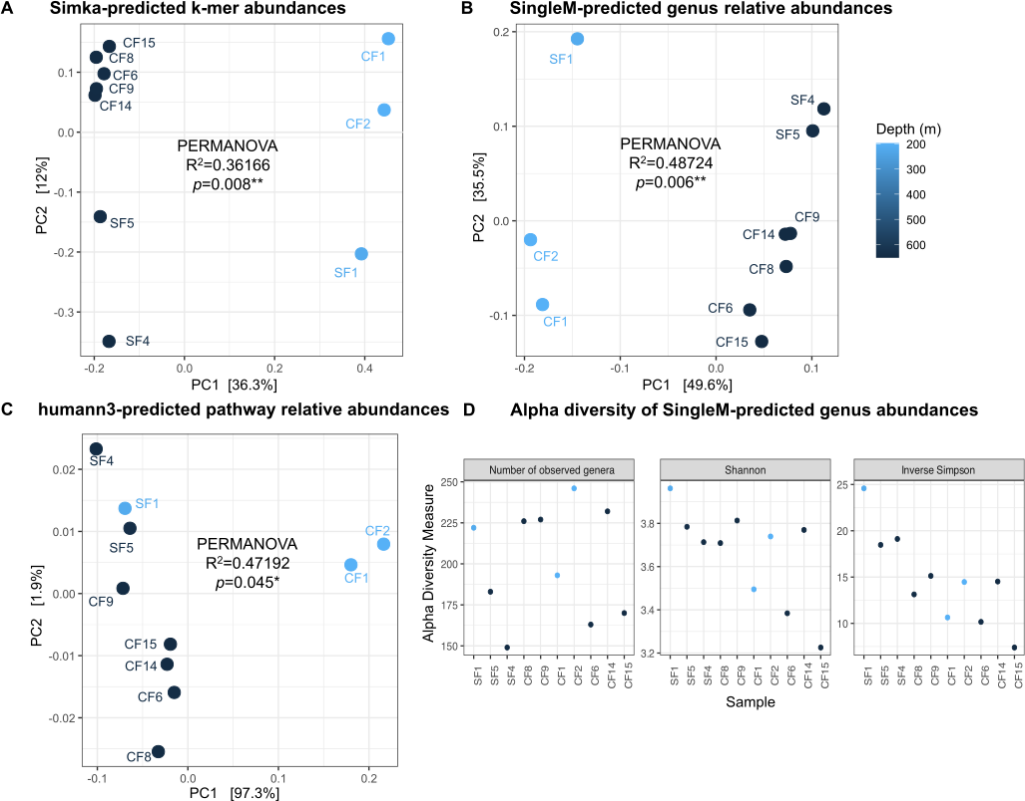
Microbial diversity differences between the 10 libraries sequenced in this study (Table 1) based on PCoA ordinations of Bray-Curtis dissimilarities computed from (**A**) *k*-mer counts predicted by Simka; (**B**) genus relative abundances predicted by SingleM; (**C**) MetaCyc pathway relative abundances predicted by HUMAnN3; and (**D**) alpha diversity indices, including the number of observed genera, Shannon index, and Inverse Simpson indices, computed from the genus relative abundance table generated by SingleM. Samples are colored by depth.

Metagenomic sequencing yielded a total of 61 Gbp from two Illumina HiSeq 2000 lanes with an average of 30 ± 4 million paired-end reads per library from the 10 samples collected in the GC233 lease area prior to the DWH oil spill (Table S1). Two distinct clusters comprising epipelagic (198–217 m) and mesopelagic samples (640–655 m) were observed on the Principal Coordinate Analysis (PCoA) plot based on the Bray-Curtis dissimilarity of the *k*-mer counts between samples (Fig. 2A; PERMANOVA *P* = 0.008) and based on the Bray-Curtis dissimilarities of the genus abundances between samples (PERMANOVA *P* = 0.005, Fig. 2B). Overall, the observed differences (Fig. 2) were consistent with other studies (21, 22) that showed distinct microbial community compositions between shallower (≤100 m) and deeper waters prior to the DWH spill (21, 22). The alpha diversity, calculated based on the genus relative abundance table generated by SingleM, did not significantly differ between the epipelagic and mesopelagic samples (Fig. 2D).

**Fig 3:**
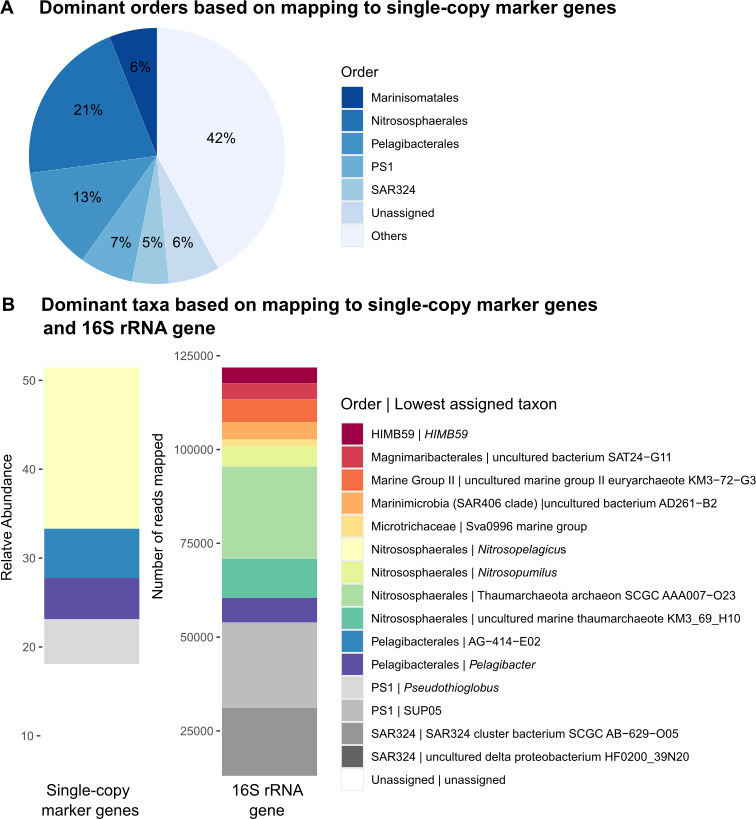
Dominant orders (**A**) and taxa (**B**; left) based on mapping to single-copy marker genes with >5% relative abundance (Data Set S1; Tables S2 and S3) and dominant taxa (**B**; right) with >4,000 reads to assembled full-length 16S rRNA gene sequences (Data Set S2; Table S4) across the 10 libraries in this study. See the supplemental material for full details. Taxonomies updated to GTDB as possible.

Taxonomy was characterized by mapping metagenomic reads across all 10 libraries to (i) single-copy archaeal and bacterial gene markers, (ii) assembled full-length 16S rRNA gene sequences, and (iii) assembling and binning reads into MAGs. Combining these analyses, predominant taxa (excluding unclassified) included three archaeal and 14 bacterial classes (Fig. 3), with details provided below for each analysis method. These taxa were assigned to phyla Acidobacteriota, Actinomycetota, Bacillota, Bacteroidota, Chloroflexota, Fidelibacterota, Myxococcota, Nitrospinota, Planctomycetota, Poribacteria, Pseudomonadota, SAR324, Thermoplasmatota, Thermoproteota, and Verrucomicrobiota.

### Mapping to single-copy marker genes

Taxa classified under the domain Bacteria had the highest average relative abundance (73% ± 5%) across libraries, followed by Archaea (27% ± 5%; Data Set S1). Abundant phyla were split between bacterial and archaeal annotations, where Pseudomonadota had the highest relative abundance (37% ± 2%) across libraries, followed by Thermoproteota (21% ± 6%), Fidelibacterota/Marinisomatota (8% ± 2%), Thermoplasmatota (5% ± 2%), SAR324 (5% ± 1%), and Actinomycetota (5% ± 1%) (Table S2). At the class level, top reads were divided between Nitrososphaeria (21% ± 6%), Alphaproteobacteria (19% ± 4%), Gammaproteobacteria (18% ± 3%), Marinisomatia (8% ± 2%), Poseidoniia (5% ± 2%), and SAR324 (5% ± 1%) (Table S2). The archaeal order Nitrososphaerales was the most abundant (21% ± 6%), followed by Pelagibacterales (13% ± 3%), PS1 (7% ± 3%), unassigned (6% ± 1%), Marinisomatales (6% ± 2%), and SAR324 (5% ± 1%; Fig. 3A). At the family level, Nitrosopumilaceae was the most abundant (21% ± 6%), followed by Pelagibacteraceae (13% ± 3%), unassigned (10% ± 2%), and Thalassarchaeaceae (4% ± 2%). The archaeal genus *Nitrosopelagicus* was most abundant among the identified genera, with 18% ± 6% average relative abundance across libraries (Fig. 3B). This is followed by unassigned genera (18% ± 2%), AG-414-E02 (6% ± 1%), and *Pelagibacter* (5% ± 1%) from class Alphaproteobacteria and *Pseudothioglobus* from class Gammaproteobacteria (5% ± 2%) (Fig. 3B; Table S3).

### 16S rRNA gene assembly

PhyloFlash (46) assembled 932 bacterial, 205 archaeal, and 44 eukaryotic full-length small subunit (SSU) rRNA sequences (Data Set S2). The top two phyla were split between the same bacterial and archaeal classifications as observed for singleM annotations, and unclassified taxa started to dominate at the level of order. Sequences with >10,000 total reads summed across the libraries included genus-level annotations within families Microtrichaceae (all Sva0996 marine group with 93.5%–99.8% sequence identities), Pseudothioglobaceae (all SUP05 cluster formerly classified under *Thioglobaceae* with 90.0%–99.5% sequence identities), and the archaeal Nitrosopumilaceae (three classifications with 88.9%–99.9% sequence identities). Predominant orders included HIMB59 (formerly linked to Rhodospirillales), Pelagibacterales (including SAR11), Pseudomonadales (including SAR86), and Marine Group II of the archaeal class Thermoplasmata (Fig. 3B). Annotations to clades SAR406 and SAR324 also predominated (Fig. 3B; Table S4). Classes Gammaproteobacteria, Alphaproteobacteria, Nitrososphaeria, Thermoplasmata, Acidimicrobiia, Nitrospinia, Bacteroidia, Verrucomicrobiae, and Dehalococcoidia were also well represented, with each receiving >3,000 total reads. Except for *NB1-j*, all phyla (Data Set S2) matched those identified from the archaeal and bacterial single-copy marker gene sequences (Data Set S1).

### Metagenome co-assembly

The metagenome co-assembled from the samples (Table 1) contained 269,466 contigs totaling 530 Mbp in size. These contigs were binned into 60 bacterial and 11 archaeal MAGs. The bacterial MAGs were identified as belonging to the phyla Acidobacteriota, Actinomycetota, Bacteroidota, Chloroflexota, Fidelibacterota, Myxococcota_A, Nitrospinota, Planctomycetota, Poribacteria, Pseudomonadota, SAR324, and Verrucomicrobiota (Table S5; Data Set S3). Taxonomic assignments of 24 MAGs matched those provided by single-copy marker genes (Data Set S1) and full-length 16S rRNA gene sequences (Data Set S2), including unclassified genera assigned to the family Thioglobaceae (*n* = 3), phylum/class/order SAR324 (*n* = 3), order Acidimicrobiales (*n* = 8), family Verrucomicrobiae (*n* = 1), and class Marinisomatia (*n* = 9). From the 11 archaeal MAGs, the phyla Thermoplasmatota and Thermoproteota were recovered (Table S5). This included one MAG assigned to an unclassified species under the genus *Nitrosopelagicus*, whose single-copy marker gene was the most abundant in the libraries at the genus level (Data Set S1).

### Analysis of functional traits

Functional annotation was used to investigate hydrocarbon biodegradation potential. A total of 2,552 MetaCyc (61) pathways were predicted by HUMAnN 3.0 (60) (supplemental material). MetaCyc pathway abundances differed across epipelagic and mesopelagic water samples (*P* = 0.045) based on Bray-Curtis dissimilarities (Fig. 2C). Further in-depth functional annotations using a variety of software revealed annotations for halogenated compound utilization, hydrocarbon degradation, and methane metabolism, with all bins ascribed to class Gammaproteobacteria (Table 2; Data Set S4).

**TABLE 2:** Linkages between taxonomic and functional annotations for hydrocarbon pathway categories^*a*^

Class and phylum	Halogenated compound utilization	Hydrocarbon degradation	Methane metabolism	C1 metabolism
Acidobacteriota				1
UBA890				1
Actinomycetota				6
Acidimicrobiia				6
Chloroflexota				7
Dehalococcoidia				7
Myxococcota_A				3
UBA9160				3
Nitrospinota				1
Nitrospinia				1
Planctomycetota				4
Planctomycetia				3
UBA8108				1
Poribacteria				1
WGA-4E				1
Pseudomonadota	2	6	5	53
Alphaproteobacteria				12
Gammaproteobacteria	2	6	5	41
SAR324				7
SAR324				7
Thermoplasmatota				3
Poseidoniia				3

^*a*^ See the supplemental material and Data Set S4 for further details.

All annotations for the particulate methane monooxygenase (*pmo*) genes involved in methane metabolism were ascribed to either order Methylococcales (*UBA1147 sp024958995* with annotations to *pmoA*, *pmoB*, and *pmoC*) or Pseudomonadales (unclassified *UBA9659* with annotations to *pmoA* and *pmoB*) (Table 2; Table S6; Data Set S4; supplemental material). The category for oxidation of formaldehyde, formate, and methanol (termed C1 metabolism in Data Set S4) contained annotations to multiple taxa. In addition to Gammaproteobacteria, class Alphaproteobacteria and nine other phyla were categorized (Table 2; Tables S7 and S8; Data Set S4, see supplemental material for detailed analysis). Overall, the mean coverage of genes associated with C_1_-compound oxidation was lower in the three shallower water samples (198–217 m; Table 1; Table S1) compared to the seven deeper samples (640–652 m, *P* < 2.2 × 10^−16^; Fig. S1). Additional comparisons of gene coverage between samples are provided in the supplemental material (Fig. S1 and S2).

Genes for the breakdown of alkane, alkene, and halogenated compounds were identified in several MAGs assigned to order Pseudomonadales (Data Set S4). For cyclic alkane degradation, two sequences partially encoding cyclohexanone monooxygenases (CHMO) were annotated in Bin10_008 classified as *UBA11889 sp002719135* and in Bin03_051 assigned to *HTCC2207 sp012960115*. The *alkB* gene for alkane 1-monooxygenase and other genes encoding an alkene reductase and haloalkane dehalogenase (*linB*) were identified in Bin05_012, assigned to an unclassified species under the genus *Marinobacter*.

**Fig 4:**
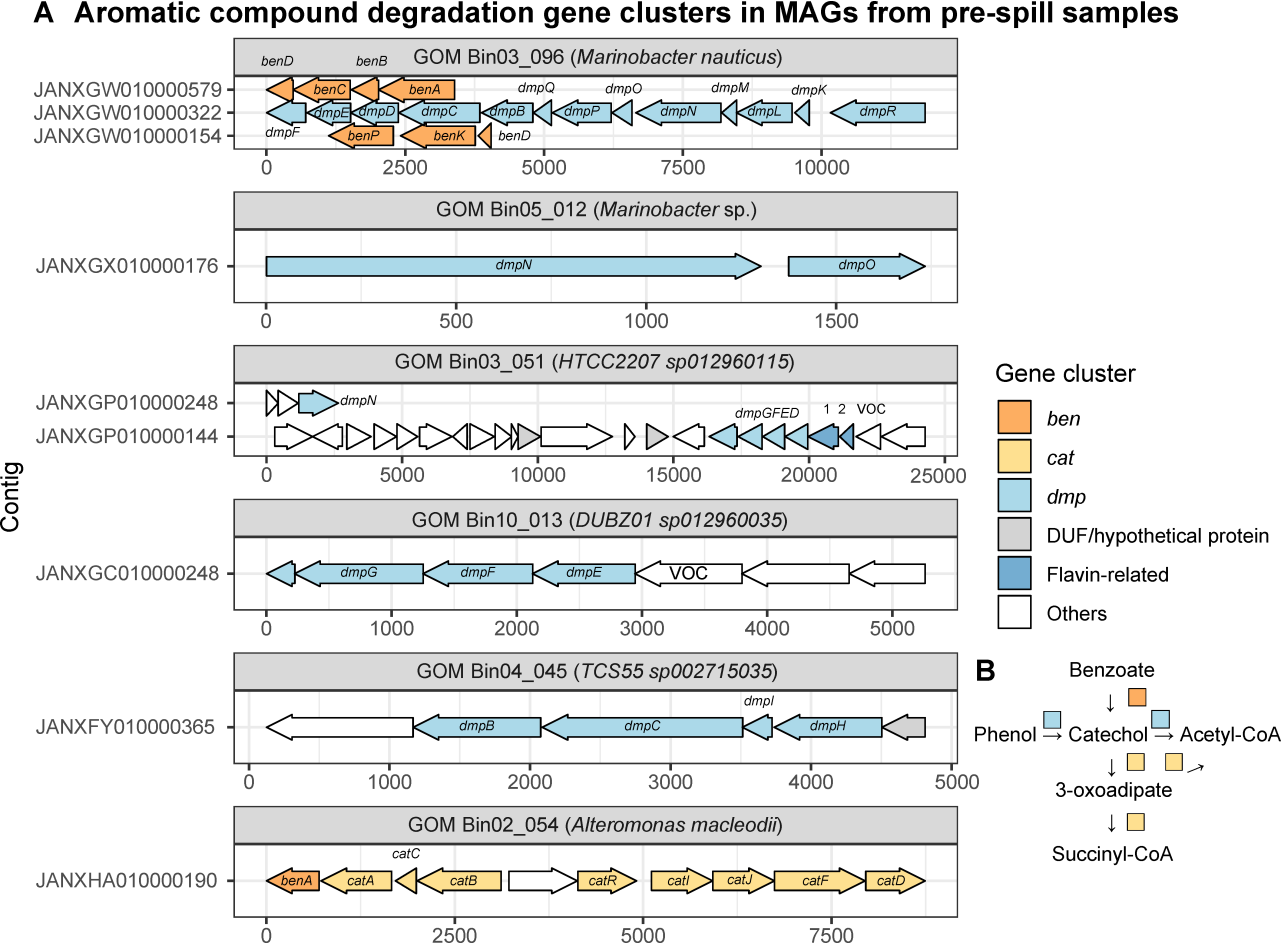
(**A**) Aromatic compound degradation genes and (**B**) overview of aromatic compound degradation pathways identified in MAGs recovered from the samples listed in Table 1. Numbered genes: 1, flavin-dependent monooxygenase; 2, flavin reductase family protein. Abbreviations: VOC, vicinal oxygen chelate family protein; DUF, domain of unknown function. Names for other unlabeled genes are in Data Set S4.

Aromatic compound degradation genes were annotated in both gammaproteobacterial and non-gammaproteobacterial MAGs (Fig. 4; Data Set S4). For gammaproteobacterial MAGs, the *benABCD* and *benKE* operons linked to the degradation of benzoate to catechol (71, 72) were identified in Bin03_096 classified as *Marinobacter nauticus* under order Pseudomonadales, with higher mean coverage for samples collected at >600 m depth in the vicinity of Brine Pool NR-1 (Fig. S2). The *benA* gene encoding the alpha subunit of benzoate 1,2-dioxygenase adjacent to the *catACBRIJFD* gene cluster was identified in Bin02_054 classified as *Alteromonas macleodii* under order Enterobacterales (Fig. 4). These enzymes degrade benzoate/3-chlorobenzoate and catechol into tricarboxylic acid (TCA) cycle intermediates via 3-oxoadipate (73, 74).

In addition to *ben* genes, other genes for catechol degradation were identified. A subset of genes from the *dmp* operon previously reported in *Pseudomonas sp. CF600* (75) that encodes genes for dimethyl phenol degradation were identified in Bin03_096 assigned to *Marinobacter nauticus*, including *dmpKLMNOP* for the ortho-hydroxylation of phenol to catechol and *dmpBCDEF* for the subsequent degradation of catechol to acetyl-CoA. Partial *dmp* operons also were identified in two other MAGs assigned to the order Pseudomonadales. Bin05_012, assigned to *Marinobacter* spp., contained a partial *dmpNO* gene cluster encoding the P3 and P4 oxygenase components of phenol hydroxylase. Bin03_051 (*HTCC2207 sp01296011*) contained *dmpN* and a 24,277-bp gene cluster that included genes encoding a vicinal oxygen chelate (VOC) family protein with catechol degradation potential (76), flavin reductase family protein, flavin-dependent monooxygenase with alkane degradation potential (28), and *dmpDEFG* genes for catechol degradation (Fig. 4). For non-gammaproteobacterial MAGs (Table S5), genes encoding the VOC family protein and *dmpEFGD* were identified in Bin10_013 (*DUBZ01 sp012960035*) assigned to order *UBA9160* under phylum Myxococcota*_A*. Genes *dmpBC* and *dmpIH* were identified in Bin04_045 (*TCS55 sp002715035*) assigned to order Marinisomatales under phylum Fidelibacterota (Data Set S4).

### Search for DWH responders in metagenomic libraries

We compared Gammaproteobacteria sequences and taxonomy in our libraries to uncultured Pseudomonadales clones isolated from the spill plume (26) and to four MAGs assigned to species *Bermanella sp913054445* by GTDB, which contained sequences matching those clones (28, 57). Although our reads abundantly mapped to gammaproteobacterial 16S rRNA gene sequences (supplemental material), none matched sequences reported for the Pseudomonadales clones (26). Although 10 of our MAGs were assigned to order Pseudomonadales (Table S5), none were assigned to genus *Bermanella*. To further investigate, we performed read recruitment using our libraries and a comprehensive collection of 55 other libraries sequenced from Gulf water samples (Table S5) to the five *Bermanella* MAGs detailed in Materials and Methods.

**Fig 5:**
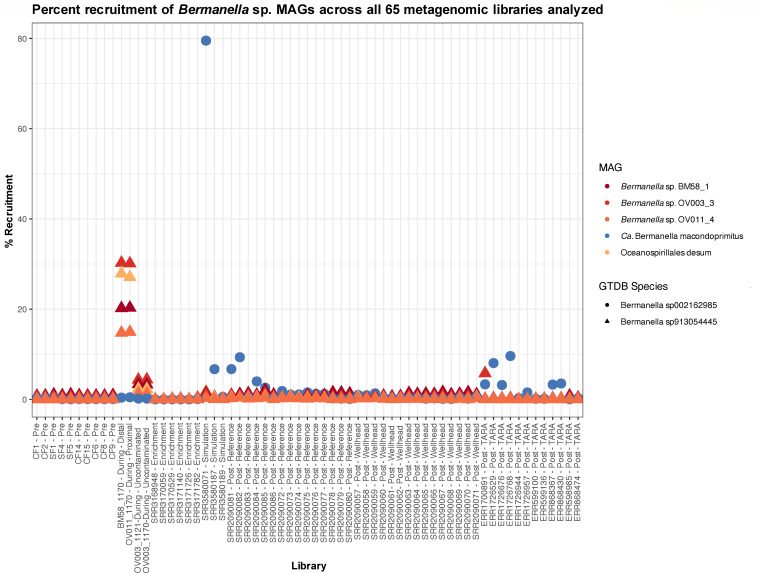
Percent recruitment of the five previously published gammaproteobacterial MAGs classified by GTDB-Tk under the genus *Bermanella* in all 65 metagenomic libraries analyzed. Percent recruitment values are calculated by anvi’o as the percentage of reads from each library that mapped to contigs in each MAG, out of all mapped reads for that specific library. The full list of percent recruitment values and NCBI accession numbers for the gammaproteobacterial MAGs are in Data Set S3. Metadata for the metagenomic libraries are in Table 1 and Table S1.

For *Bermanella sp913054445*, highest recruitment was observed for environmental samples collected during the DWH event. Highest recruitment was observed for samples from DWH plume stations (BM58_1170 = 23% ± 7%; OV011_1170 = 19% ± 7%), followed by a station outside the plume (OV003 = 3% ± 1%) (24) (Fig. 5; Data Set S3). Recruitment was low for all other analyzed libraries, with values of 0.3% ± 0.4% for this study; 0.5% ± 0.4% in samples collected 1 year after the DWH spill near the wellhead, and 0.6% ± 0.4% for reference station A6 away from the wellhead (30); 0.2% ± 0.8% from *Tara* station 142 2 years after the spill (29); and 0.2% ±0.4% in experimental samples (31, 32).

Besides *Bermanella sp913054445*, another MAG classified as *Bermanella sp002162985* was assembled from Hu et al.’s simulation (32). The 16S rRNA gene of this MAG did not match the Pseudomonadales clones isolated from the spill plume (26). Similarly, recruitment of this MAG was low for DWH plume samples (0.4% BM58_1170, 0.46% OV011_1170), our libraries (0.1% ± 0.2%), and samples collected 1 year after the DWH spill near the wellhead (0.4% ± 0.3%). Recruitment values were higher (2% ± 3%) for reference station A6 located away from the wellhead 1 year after the spill (30) and for *Tara* station 142 (3% ± 3%) collected 2 years after the spill (29). Recruitment was high (79%) for day 6 of the Hu et al. experiment (32); however, recruitment was only 0.3% ± 1% in other libraries sequenced from simulation and experimental samples (31, 32).

To explore potential reservoirs of *Bermanella* spp. in the global ocean, we performed a phylogenomic analysis of all *Bermanella* genomes found in the NCBI Genome database (Fig. S3). *Bermanella* sp. MAG MOW.maxbin2.006 assembled from a hydrothermal plume sample collected from the Guaymas Basin in 2021 (77) was the closest identified genome relative to *Bermanella sp913054445* and *Bermanella sp002162985*. These genomes formed a sister clade with two *Bermanella* spp. MAGs assembled from a seawater sample collected from Canada in 2013 (NCBI genome accession GCA_913058575) and an oil enrichment culture of a sediment sample collected from the Barents Sea in the Arctic Ocean in 2020 (NCBI genome accession GCA_036960565) (Fig. S3).

## DISCUSSION

This study provides insight into the microbial taxonomy and function of samples collected from the Gulf of Mexico Green Canyon lease area prior to the DWH spill. Oil production was active during sample collection, and spills in Green Canyon have occurred, including an 88,200-gallon spill in 2016 that resulted in a $3.9 million settlement (https://incidentnews.noaa.gov/incident/9277). Although collected outside the defined DWH plume area, the microbial potential of these samples is of general interest because baseline information has primarily been limited to amplicon sequencing (18, 21–23). Whether the DWH-oiled area has returned to baseline remains an active question (3); such knowledge is needed for impact assessments and the development of effective strategies for disaster mitigation, bioremediation, and habitat conservation (1, 2).

We surveyed microbial composition of these samples based on the mapping of metagenomic reads to single-copy marker genes, assembly of full-length 16S rRNA gene sequences, and functional annotation of MAG sequences with taxonomic linkages. Abundant sequence assignments included the archaeal genus *Nitrosopelagicus* within the family Nitrosopumilaceae (Fig. 3B; Data Set S1 and Data Set S2), which comprises ammonia-oxidizing chemolithoautotrophs that are abundant in marine and terrestrial ecosystems (78). *Nitrosopelagicus* was previously sequenced from the open ocean (79) and reported to be abundant in the Gulf at >100 m depth before the DWH oil spill (22).

Genera abundant in these samples matched reports from pre-spill Gulf samples collected at >100 m depths, including members of Actinomycetota, Alphaproteobacteria, Gammaproteobacteria, SAR324, and Verrucomicrobiota (18, 22, 80). Taking a closer look at specific abundant sequences, highest read recruitment was to the 16S rRNA gene sequence of *Thaumarchaeota archaeon SCGC AAA007-O23* (GenBank accession ARWO01000004; now classified as *Nitrosopelagicus sp000402075*) previously assembled from single cells obtained from the Atlantic Ocean (81), with 88.9%–99.7% sequence identity and 24,672 total mapped reads summed across the 10 libraries. This was followed by recruitment to an uncultured bacterium from the family Thioglobaceae and SUP05 clade (GenBank accession HQ674272) sequenced from the Northeast subarctic Pacific Ocean at 1,000 m depth (82), with 92.5%–96.8% sequence identity and 22,738 total reads (Table S4). Next highest recruitment was to sequences within the SAR324 clade at the level of class (bacterium SCGC AB-629-O05, GenBank accession AQVX01000192; sample source unknown) with 18,148 total reads and 97.1%–99.9% sequence identity. This was followed by 13,029 reads mapped to sequences 92.9%–99.7% identical to the 16S rRNA gene sequence of an uncultured delta proteobacterium HF0200_39N20 (GenBank accession GU474883) from a water sample collected at the Hawaii Oceanographic time-series study site ALOHA (83). High recruitment was observed (10,407 total reads, 99%–99.9% identity) to the uncultured marine thaumarchaeote KM3_69_H10 under phylum Nitrososphaerota (GenBank accession KF901022) sequenced from Mediterranean waters at 3,000 m depth (84). The most abundant eukaryotic SSU rRNA sequences shared 98.2%–99.5% identity to the eukaryotic 18S rRNA gene sequenced from the hydrothermal vent mussel *Adipicola* sp. collected from Juan de Fuca Ridge in the Northeast Pacific Ocean (85), with 9,242 total mapped reads (Data Set S2).

Rare members of the microbiome can respond during an oil spill, and the community, as a whole, executes biodegradation with genes from a single pathway distributed across multiple taxa (3) to help attenuate the adverse effects of oil spills on biodiversity (86). Microbial diversity surveys performed by the Gulf of Mexico Research Consortium (Consorcio de Investigación del Golfo de México [CIGoM]) from March 2015 to September 2017 revealed the dominance of *Alteromonas* in the southern Gulf water column a few years after the DWH oil spill (2). *Alteromonas* species are capable of aromatic hydrocarbon degradation and may contribute to oil dispersion in the event of a spill (2). Our data set contained single-copy marker gene and 16S rRNA gene sequences matching those from *Alteromonas*, as well as a MAG classified as *A. macleodii* with catechol degradation potential. Other taxa with hydrocarbon-degrading potential identified in the CIGoM data set, such as *Alcanivorax*, *Acinetobacter*, *Halomonas*, *Pseudoalteromonas*, and *Pseudomonas* (2), were detected in our singleM data set (Data Set S1), but not in the 16S rRNA gene data set (except for *Pseudoalteromonas*; Data Set S2) or assembled MAGs (Data Set S3). The CIGoM samples were collected from the Mexican territory and spatial variations in bacterial communities were observed even within the CIGOM water column samples (2). On a larger spatiotemporal scale, differences in environmental conditions could influence microbial communities and bioremediation response in the Gulf, and these differences should be further characterized to understand and model microbial responses to hydrocarbon exposures.

In our metagenomes, hydrocarbon degradation genes from various taxa were annotated in three main categories: (i) methane monooxygenase, methanol and ethanol dehydrogenase, and formaldehyde and formate dehydrogenase found in gammaproteobacterial MAGs; (ii) alkane, alkene, and halogenated compound degradation, found in gammaproteobacterial MAGs; (iii) aromatic compound (including benzoate, phenol, and catechol) degradation, found in both gammaproteobacterial and non-gammaproteobacterial MAGs (Data Set S4). These hydrocarbon degradation genes are not exclusive to oil and gas reservoirs. Methylotrophs with similar one-carbon metabolism pathways are common in coastal and deep sea habitats (87). Although alkane degradation was reported to be the most abundant hydrocarbon degradation pathway in the DWH plume (28), a comprehensive analysis of MAGs recovered from the global ocean also revealed the prevalence and diversity of alkane degradation genes, such as *alkB*, in broad-spectrum hydrocarbon-degrading bacteria from phyla Proteobacteria and Actinobacteriota (88). The ubiquity of alkane degradation was hypothesized to be linked to cyanobacterial alkane biosynthesis in the global ocean (88). In comparison, aromatic compound degradation genes were less diverse in the global ocean, although genes such as *dmpO* were commonly detected in Pseudomonadaceae (88). Several hydrocarbon degradation genes, such as methane monooxygenase (89), alkane 1-monooxygenase (*alkB*) (90), and haloalkane dehalogenase (91), have been studied for their applicability in the bioremediation of hydrocarbon-impacted environments. That said, gene presence does not confirm enzymatic activity, and *in situ* bioremediation capacity needs further evaluation.

In addition to characterizing microbiome diversity and function in these historical samples, sequences were investigated for rare biosphere members that dominated the early phase of the DWH spill microbial succession, namely uncultured Oceanospirillales/Pseudomonadales later classified as *Bermanella* spp. (26, 28, 57). Although several taxa from the order Pseudomonadales were identified in our data sets (Fig. 3; supplemental material and Data Set S2), no sequences were similar to or classified as *Bermanella* spp. in our metagenomic libraries, MAGs, or assembled full-length 16S rRNA gene sequences. Expanding this search by recruitment analysis revealed some read mapping for our libraries and others (Table S1). Recruitment to MAGs classified as *Bermanella sp913054445* (28, 57) was <1% for our libraries, libraries sequenced after the spill, and experimental libraries (Data Set S3; Fig. 5). Recruitment to the *Ca*. Bermanella macondoprimitus MAG classified as *Bermanella sp002162985* assembled from experimental samples and simulation/enrichment experiments (32) was <1% for most samples, and 2%–3% for samples collected at reference station A6 (30) and *Tara* station 142 (29) after the spill. Although the range of recruitment for our libraries was similar to several others, detection may have been affected by sequencing depth and platform bias, and extrapolating presence/absence patterns from single time-point metagenomes to seed bank function is uncertain. Future investigation into the diversity, environmental reservoirs, and ecological roles of *Bermanella* spp. is warranted.

Overall, this study characterizes microbiome diversity and function in historical samples from the Green Canyon area, providing missing baseline data that can aid machine learning. Furthermore, it offers insights into the diversity and potential reservoir of *Bermanella* spp. in the broader Gulf and global ocean to aid in understanding natural bioremediation capacity.

## Data Availability

Sequenced reads, metagenomes, and MAG assemblies from our samples (Table 1) were deposited in NCBI under the BioProject ID PRJNA870083. Accession numbers of reads sequenced from other studies are provided in Table S1.

## SUPPLEMENTAL MATERIAL

Data Set S1 aem.00799-26-s0001.xlsx Relative abundance and taxonomy of each OTU.

Data Set S2 aem.00799-26-s0002.xlsx 16S rRNA gene assembly results by PhyloFlash.

Data Set S3 aem.00799-26-s0003.xlsx Metagenome assembly.

Data Set S4 aem.00799-26-s0004.xlsx Functional annotations and mean coverage.

Supplemental material aem.00799-26-s0005.pdf Supplemental methods and results, Tables S1 to S8, and Fig. S1 to S3.

ASM does not own the copyrights to Supplemental Material that may be linked to, or accessed through, an article. The authors have granted ASM a non-exclusive, world-wide license to publish the Supplemental Material files. Please contact the corresponding author directly for reuse.
