# Supplementary material for: Metagenomic analysis of water column samples collected from Green Canyon 233 prior to the Deepwater Horizon incident
Source: Appl Environ Microbiol. 2026 Jun 18;92(7):e00799-26. doi: 10.1128/aem.00799-26 (PMC13390413; doi:10.1128/aem.00799-26)
Supplement: Supplemental material — Supplemental methods and results, Tables S1 to S8, and Fig. S1 to S3. [file aem.00799-26-s0005.pdf]

SUPPLEMENTAL MATERIAL

Metagenomic analysis of water column samples collected from Green Canyon 233 prior to the Deepwater Horizon incident

SUPPLEMENTAL METHODS

Gene coverage analysis

Coverage data for all genes associated with contigs in the co-assembled metagenome from the 10 libraries sequenced for this study were exported from the contigs database in anvi'o (1) using the anvi-export-gene-coverage-and-detection command. Coverage data for hydrocarbon degradation genes were extracted from the exported coverage dataset using a bash script. The distribution of coverage values for hydrocarbon degradation genes was tested for normality using the Shapiro-Wilk test (2), which rejected the null hypothesis that the data was normally distributed ($p<2.2x10^{-6}$). Pairwise comparisons between sample groups were performed for each subset of hydrocarbon degradation gene using the non-parametric Mann–Whitney U test (3) using the wilcox.test function implemented in R.

SUPPLEMENTAL RESULTS

Functional annotations of the metagenomic libraries of the historical samples

To investigate the bioremediation potential to degrade hydrocarbons, sequences were functionally annotated. A total of 2,552 MetaCyc (32) pathways were predicted by HUMAnN 3.0 (4) for the ten metagenomic libraries sequenced in this study (**Table 1**). Pathways predicted under the class "aromatic compound degradation" included 2-aminophenol degradation, 3-phenylpropanoate and 3-(3-hydroxyphenyl)propanoate degradation, 3-phenylpropanoate and 3-(3-hydroxyphenyl)propanoate degradation to 2-hydroxypentadienoate, 4-methylcatechol

degradation (ortho cleavage), biphenyl degradation, and the superpathway of salicylate degradation.

In-depth functional annotation with taxonomic linkages

In-depth functional annotations (**Data Set S4**) employed a variety of software (anvi'o, METABOLIC, and PGAP, and verified by web blast searches). This analysis returned four categories for hydrocarbon pathways: halogenated compound utilization, hydrocarbon degradation, methane metabolism, and C1 metabolism. Except for C1 metabolism, all annotations were classified under phylum Pseudomonadota and class Gammaproteobacteria (**Table 3**). The halogenated utilization compound category contained a single function description termed halogenated compounds breakdown. This function annotated a single gene (haloalkane dehalogenase, *linB*) with taxonomic classifications to order Pseudomonadales -- family Porticoccaceae (HTCC2207 sp012960115) and family Oleiphilaceae (unclassified *Marinobacter*) (**Data Set S4**, **Table S7**). The hydrocarbon degradation category contained three descriptions of function: alkane degradation, cyclic alkane degradation, and alkene reduction. Each function was annotated by a single gene: alkane 1-monooxygenase (*alkB*), cyclohexanone monooxygenase (*CHMO*), and alkene reductase (*ER*), respectively. All annotations to these functions were ascribed to order Pseudomonadales -- family Azotimanducaceae_*A* (UBA11889 sp002719135), family Oleiphilaceae (unclassified *Marinobacter*), and family Porticoccaceae (HTCC2207 sp012960115) (**Data Set S4**; **Table S7**).

The methane metabolism category contained a single description of function termed methane oxidation - particulate methane monooxygenase that was annotated by three methane/ammonia monooxygenase genes (*pmoA*, *pmoB*, and *pmoC*). All annotations were

ascribed to either order Methylococcales or Pseudomonadales (**Data Set S4**, **Table S7**). The C1 metabolism category contained three descriptions of function: formaldehyde oxidation, formate oxidation, and methanol oxidation. The majority of classifications were to phylum Pseudomonadota, with annotations to both Gammaproteobacteria and Alphaproteobacteria classes (**Table S7**). In addition, annotations were attributed to phyla Acidobacteriota, Actinomycetota, Chloroflexota, Myxococcota_A, Nitrospinota, Planctomycetota, Poribacteria, SAR324, and Thermoplasmatota (**Table S8**). Formaldehyde oxidation was annotated by four genes, formate oxidation by three genes, and methanol oxidation by one gene. Annotations were distributed across multiple taxa (**Data Set S4**), with further details provided below.

C1-compound oxidation genes

Genes for the oxidation of gaseous one-carbon compounds were predicted in MAGs classified under the class Gammaproteobacteria, whose members are known to cycle C_1-compounds (78). Genes encoding methane monooxygenase/ammonia monooxygenase (*pmoABC*) were completely sequenced in Bin06_002, which was classified by GTDB-Tk (58) (**Data Set S3**) as *UBA1147 sp024958995* (order: Methylococcales) and partially sequenced (*pmoAB*) in Bin02_118 classified as *UBA9659* sp. (order: Pseudomonadales). Sequences partially encoding a pyrroloquinoline quinone (PQQ)-dependent methanol/ethanol dehydrogenase and formaldehyde-activating enzymes were identified in Bin06_002 classified as *UBA1147 sp024958995*. These and various formaldehyde oxidation and formate oxidation genes also were annotated in other MAGs assembled in this study.

Homologs of the pyrroloquinoline quinone (PQQ)-dependent methanol/ethanol dehydrogenase protein sequence (*MxaF*) used by methylotrophs to methanotrophs were

detected in gammaproteobacterial and non-gammaproteobacterial MAGs (**Data Set S4**). Gammaproteobacterial MAGs with this protein-coding gene were mostly classified by GTDB-Tk (6) under the order Pseudomonadales and included Bin01_161 (*ASP10-02a* unclassified), Bin03_051 (*HTCC2207 sp012960115*), Bin06_002 (*UBA1147 sp024958995*), Bin10_008 (*UBA11889 sp002719135*), Bin04_082 (*UBA11889 unclassified*), and Bin02_020 (*UBA9145 sp002694855*). Other gammaproteobacterial MAGs containing this gene included Bin02_011 classified as *AEGEAN-183 sp012965075* under the order *SAR86*, Bin02_054 classified as *Alteromonas macleodii* under the order Enterobacterales, and Bin03_069 assigned to an unclassified species under the genus *UBA11869* and order *UBA4486*. Non-gammaproteobacterial MAGs that contained genes for the PQQ-dependent methanol/ethanol dehydrogenase included Bin06_029 (*Paracoccus sp002294185*), Bin01_094 (*UBA890 sp002722645*), Bin01_175 (*UBA9662 sp002697225*), and Bin10_026 (*JAZXM01* unclassified).

Formaldehyde oxidation genes were detected in some MAGs with methanol/ethanol oxidation potential. For glutathione-dependent formaldehyde detoxification, sequences encoding S-formylglutathione hydrolase were annotated in Bin01_161 (*ASP10-02a* unclassified), Bin02_054 (*Alteromonas macleodii*), Bin03_051 (*HTCC2207 sp012960115*), and Bin03_096 (*Marinobacter nauticus*). Sequences encoding S-(hydroxymethyl)glutathione dehydrogenase/class III alcohol dehydrogenase similarly associated with glutathione-dependent formaldehyde oxidation were detected in Bin02_011 (*AEGEAN-183 sp012965075*), Bin03_051 (*HTCC2207 sp012960115*), Bin03_096 (*Marinobacter nauticus*), Bin04_082 (*UBA11889 unclassified*), Bin02_020 (*UBA9145 sp002694855*), Bin06_029 (*Paracoccus sp002294185*), and Bin10_026 (*JAZXM01* unclassified).

Genes involved in formaldehyde oxidation were also annotated in other MAGs with no sequenced methanol/ethanol oxidation potential. Sequences encoding the formaldehyde-activating enzyme, which adds glutathione to formaldehyde to form S-hydroxymethylglutathione, were mostly annotated in MAGs classified under the phylum Planctomycetota, including Bin01_182 (*QWPN01 sp027592945*), Bin02_043 (*UBA721* unclassified), and Bin03_093 (UBA8108 unclassified), except for gammaproteobacterial MAG (*UBA1147 sp024958995*). Genes encoding both S-formylglutathione hydrolase and S-(hydroxymethyl)glutathione dehydrogenase/class III alcohol dehydrogenase were identified in Bin03_022 (*Arctic96AD-7 sp022448485*), Bin04_031 (*DUCF01* unclassified), Bin01_230 (*UBA2705 sp002690045*), Bin01_180 (*UBA9659 unclassified*). Other MAGs encoded only sequences for S-(hydroxymethyl)glutathione dehydrogenase/class III alcohol dehydrogenase, including Bin01_114 (*GCA-2731375 sp913049615*), Bin03_002 (*Arctic96AD-7 sp002082305*), and Bin10_013 (*DUBZ01 sp012960035*). Sequences encoding S-(hydroxymethyl)mycothiol dehydrogenase, which oxidizes S-hydroxymethylmycothiol formed spontaneously from formaldehyde and mycothiol, were mostly identified in MAGs classified under the class *Acidimicrobiia*, including Bin03_060 (S20-B6 sp002699725), Bin04_071 (*UBA2110 sp002388005*), Bin02_055 (*UBA2110 sp002719335*), Bin02_145 (*UBA2110 sp002705305*), Bin03_010 (*UBA9410 sp022452145*), Bin03_054 (*UBA9410* unclassified). Three MAGs classified under the class *Dehalococcoidia* also encoded for S-(hydroxymethyl)mycothiol dehydrogenase, including Bin04_055 (*UBA11650 sp002708395*), Bin04_052 (*UBA1328* unclassified), and Bin10_049 (*UBA9611 sp002697005*). This protein-coding gene was also annotated in another

alphaproteobacterial MAG, Bin02_064 assigned to an unclassified species under the genus *GCA-2712665*.

For formate oxidation, the formate dehydrogenase subunit alpha (*fdhF*) and formate dehydrogenase FDH3 subunit beta (*fdh3B*) genes were identified in the alphaproteobacterial MAGs Bin01_114 (*GCA-2731375 sp913049615*) and Bin06_029 (*Paracoccus sp002294185*), and Bin04_038 assigned to *UBA9611 sp002698265* under the class Dehalococcoidia. *fdhF* was also annotated in eight other gammaproteobacterial MAGs, including Bin04_031 (*DUCF01* unclassified), Bin03_015 (*HTCC2207 sp024959305*), Bin05_012 (*Marinobacter* unclassified), Bin06_002 (*UBA1147 sp024958995*), Bin04_007 (*UBA11654* sp.), Bin04_082 (*UBA11889 unclassified*), Bin02_020 (*UBA9145 sp002694855*), Bin02_118 (*UBA9659* unclassified). Other MAGs containing *fdfH* were assigned to the bacterial classes Alphaproteobacteria (Bin01_140 classified as *GCA-2731375 sp913049615*), Dehalococcoidia (Bin02_065 classified as *UBA11996 sp002435305* and Bin06_002 classified as *UBA1147 sp024958995*), Nitrospinia (Bin02_087 classified as *LS-NOB* sp.), and *SAR324* (Bin03_002 classified as *Arctic96AD-7 sp002082305* and, Bin03_022 classified as *Arctic96AD-7 sp022448485*).

Gene coverages compared across samples.

The mean coverage for genes for the breakdown of alkane, alkene, and halogenated compounds was higher in the libraries sequenced from samples collected at >600 m depth outside Brine Pool NR-1 (CF8 and CF9, 649 m; **Table 1**, **Table S1**) compared to the other eight samples, including samples collected just above the pool (p= 8.678x10^{-5}; **Fig. S1**).

The mean coverage of *ben* genes was higher in libraries sequenced from samples collected at >600 m depth in the vicinity of Brine Pool NR-1 (samples SF4, SF5, CF8, CF9, 640-

649m; **Table 1**, **Table S1**) compared to the other six samples (p=7.014x10^{11}; **Fig. S2**). The mean coverage was higher for *dmp* (p<2.2x10^{-16}) and *cat* (p=4.367x10^{-10}) genes in the libraries sequenced from ROV-collected samples (CF8 and CF9, 649 m depth) outside Brine Pool NR-1, compared to the other eight samples. For flavin reductase family protein and flavin-dependent monooxygenase genes, there was zero coverage in shallower water samples (CF1, CF2, SF1), while mean coverage in the seven other libraries was >0 (p=0.0005; **Fig. S2**).

Assembled gammaproteobacterial SSU rRNA sequences.

From the class Gammaproteobacteria, 251 assembled SSU rRNA sequences were assigned to orders Pseudomonadales (n=127), HOC36 (n=32), UBA10353 marine group (n=28), Arenicellales (n=25), Methylococcales (n=19), Enterobacterales (n=14), Steroidobacterales (n=5), and Ga0077536 (n=1). Assembled Pseudomonadales sequences matched sequences of uncultured bacteria from the KI89A clade, Marinobacteraceae, Pseudohongiellaceae, Rhodobacteraceae, the SAR86 clade (now a separate order), and Thioglobaceae.

SUPPLEMENTAL TABLES

TABLE S1 Description of all metagenomic libraries analyzed for this manuscript, including libraries sequenced from ten water samples collected for this study (**Table 1**) and from water samples collected in other studies. *Sequences from Mason *et al.* (2012) were downloaded from *http://mason.eoas.fsu.edu/DWH_plume/metagenomes/*.

Reference	Sample / accession no.	Month	Year	Sample description	Depth (m)	Depth category	Latitude (Degree)	Longitude (Degree)	Size Fraction (uM)	Total # Reads	Sequencing Method
This study	SF1 / SRX17158992	Sep	2009	Brine Pool NR-1 CTD4-01, cast 80, bottle 19 (filter SF1), ~430m above brine pool	217	Epipelagic	27.72335	-91.28117	0.2	31,372,539	Illumina HiSeq 2000 (2x100bp)
	SF5 / SRX17158993	Sep	2009	Brine Pool NR-1 CTD4-01, cast 80, bottle 3 (filter SF5), ~10m above brine pool	640	Mesopelagic	27.72335	-91.28117	0.2	29,041,914	
	SF4 / SRX17158994	Sep	2009	Brine Pool NR-1 CTD4-01, cast 80, bottle 1, just above brine pool (filter SF4)	645	Mesopelagic	27.72335	-91.28117	0.2	26,289,108	
	CF8 / SRX17158998	Sep	2009	Brine Pool NR-1 Dive #3752 ROV Niskin collected ~3m west of outer mussel edge (filter RefA)	649	Mesopelagic	27.72371	-91.27926	0.2	40,015,922	
	CF9 / SRX17158999	Sep	2009	Brine Pool NR-1 Dive #3752 ROV Niskin collected ~3m west of outer mussel edge (filter RefC)	649	Mesopelagic	27.72371	-91.27926	0.2	35,805,503	

Reference	Sample / accession no.	Month	Year	Sample description	Depth (m)	Depth category	Latitude (Degree)	Longitude (Degree)	Size Fraction (uM)	Total # Reads	Sequencing Method
	CF1 / SRX17158990	Oct	2009	CTD8-01, cast 84, bottle 11 (filter A)	198	Epipelagic	27.72353	-91.279	0.2	26,984,306	
	CF2 / SRX17158991	Oct	2009	CTD8-01, cast 84, bottle 11 (filter C)	198	Epipelagic	27.72353	-91.279	0.2	30,674,505	
	CF6 / SRX17158995	Oct	2009	CTD8-01, cast 84, bottle 2 (filter D)	643	Mesopelagic	27.72353	-91.279	0.2	28,214,888	
	CF14 / SRX17158996	Oct	2009	CTD8-01, cast 84, bottle 1 (filter A)	652	Mesopelagic	27.72353	-91.279	0.2	32,817,661	
	CF15 / SRX17158997	Oct	2009	CTD8-01, cast 84, bottle 1 (filter C)	652	Mesopelagic	27.72353	-91.279	0.2	27,174,875	
Mason *et al.* (2012) (during spill)	BM58_1170*	May	2010	Distal station (BM58)	1179	Bathypelagic	28.672323	-88.435935	0.2	71,339,076	Illumina GAIIx (2x100bp)
	OV011_1170*	May	2010	Proximal station (OV011)	1207	Bathypelagic	28.732011	-88.376789	0.2	61,090,938	
	OV003_1121*	May	2010	Uncontaminated station (OV003)	1020	Bathypelagic	28.666022	-88.756806	0.2	28,579,679	
	OV003_1170*	May	2010	Uncontaminated station (OV003)	1020	Bathypelagic	28.666022	-88.756806	0.2	60,335,450	
Yergeau *et al.* (2015) (post-spill)	SRS981310	Sep	2011	Reference (A6)	1284	Bathypelagic	28.6632	-88.0095	0.2	333,769	Ion Torrent PGM single-end reads
	SRS981311	Sep	2011	Reference (A6)	1284	Bathypelagic	28.6632	-88.0095	0.2	77,623	
	SRS981312	Sep	2011	Reference (A6)	1284	Bathypelagic	28.6632	-88.0095	0.2	1,103,740	
	SRS981307	Sep	2011	Reference (A6)	2174	Bathypelagic	28.6632	-88.0095	0.2	807,363	
	SRS981308	Sep	2011	Reference (A6)	2174	Bathypelagic	28.6632	-88.0095	0.2	497,694	
	SRS981309	Sep	2011	Reference (A6)	2174	Bathypelagic	28.6632	-88.0095	0.2	234,606	
	SRS981316	Sep	2011	Reference (A6)	100	Epipelagic	28.6632	-88.0095	0.2	669,628	
	SRS981317	Sep	2011	Reference (A6)	100	Epipelagic	28.6632	-88.0095	0.2	398,782	
	SRS981318	Sep	2011	Reference (A6)	100	Epipelagic	28.6632	-88.0095	0.2	748,135	
	SRS981319	Sep	2011	Reference (A6)	1	Epipelagic	28.6632	-88.0095	0.2	374,960	
	SRS981320	Sep	2011	Reference (A6)	1	Epipelagic	28.6632	-88.0095	0.2	358,015	
	SRS981321	Sep	2011	Reference (A6)	1	Epipelagic	28.6632	-88.0095	0.2	851,996	
	SRS981313	Sep	2011	Reference (A6)	700	Mesopelagic	28.6632	-88.0095	0.2	944,012	

Reference	Sample / accession no.	Month	Year	Sample description	Depth (m)	Depth category	Latitude (Degree)	Longitude (Degree)	Size Fraction (uM)	Total # Reads	Sequencing Method
	SRS981314	Sep	2011	Reference (A6)	700	Mesopelagic	28.6632	-88.0095	0.2	546,243	
	SRS981315	Sep	2011	Reference (A6)	700	Mesopelagic	28.6632	-88.0095	0.2	685,449	
	SRS981277	Sep	2011	Wellhead (BM-57)	100	Epipelagic	28.7051	-88.4016	0.2	799,249	
	SRS981331	Sep	2011	Wellhead (BM-57)	100	Epipelagic	28.7051	-88.4016	0.2	338,125	
	SRS981332	Sep	2011	Wellhead (BM-57)	100	Epipelagic	28.7051	-88.4016	0.2	954,547	
	SRS981333	Sep	2011	Wellhead (BM-57)	1	Epipelagic	28.7051	-88.4016	0.2	502,617	
	SRS981334	Sep	2011	Wellhead (BM-57)	1	Epipelagic	28.7051	-88.4016	0.2	867,659	
	SRS981335	Sep	2011	Wellhead (BM-57)	1	Epipelagic	28.7051	-88.4016	0.2	841,278	
	SRS981328	Sep	2011	Wellhead (BM-57)	850	Mesopelagic	28.7051	-88.4016	0.2	501,396	
	SRS981329	Sep	2011	Wellhead (BM-57)	850	Mesopelagic	28.7051	-88.4016	0.2	702,869	
	SRS981330	Sep	2011	Wellhead (BM-57)	850	Mesopelagic	28.7051	-88.4016	0.2	407,731	
	SRS981325	Sep	2011	Wellhead (BM-57)	1174	Bathypelagic	28.7051	-88.4016	0.2	812,159	
	SRS981326	Sep	2011	Wellhead (BM-57)	1174	Bathypelagic	28.7051	-88.4016	0.2	1,351,852	
	SRS981327	Sep	2011	Wellhead (BM-57)	1174	Bathypelagic	28.7051	-88.4016	0.2	1,516,826	
	SRS981322	Sep	2011	Wellhead (BM-57)	1574	Bathypelagic	28.7051	-88.4016	0.2	323,708	
	SRS981323	Sep	2011	Wellhead (BM-57)	1574	Bathypelagic	28.7051	-88.4016	0.2	1,084,610	
	SRS981324	Sep	2011	Wellhead (BM-57)	1574	Bathypelagic	28.7051	-88.4016	0.2	1,568,609	
Pesant *et al.* (2015) (post-spill)	ERR599100	Jan	2012	TARA_142_DCM_0.22-3	125	Epipelagic	25.66385	-88.4724	0.22-3	155,790,912	Illumina HiSeq 2000 (2x100bp)
	ERR868367	Jan	2012	TARA_142_DCM_0.8-5	125	Epipelagic	25.66385	-88.4724	> 0.8	164,724,099	
	ERR1726957	Jan	2012	TARA_142_DCM_180-2000	125	Epipelagic	25.58345	-88.4339	180-2000	155,981,594	
	ERR1726529	Jan	2012	TARA_142_DCM_20-180	125	Epipelagic	25.602	-88.44445	20-180	176,263,128	
	ERR1700891	Jan	2012	TARA_142_DCM_5-20	125	Epipelagic	25.66385	-88.4724	W5-20	150,389,878	
	ERR599136	Jan	2012	TARA_142_SRF_0.22-3	5	Epipelagic	25.534	-88.3992	0.22-3	157,141,812	

Reference	Sample / accession no.	Month	Year	Sample description	Depth (m)	Depth category	Latitude (Degree)	Longitude (Degree)	Size Fraction (uM)	Total # Reads	Sequencing Method
	ERR1726676	Jan	2012	TARA_142_SRF_0.8->	5	Epipelagic	25.534	-88.3992	> 0.8	170,864,142	
	ERR868430	Jan	2012	TARA_142_SRF_0.8-5	5	Epipelagic	25.534	-88.3992	> 0.8	163,847,821	
	ERR1726944	Jan	2012	TARA_142_SRF_180-2000	5	Epipelagic	25.56275	-88.4148	180-2000	180,046,742	
	ERR1726768	Jan	2012	TARA_142_SRF_20-180	5	Epipelagic	25.59135	-88.4296	20-180	138,440,102	
	ERR598985	Jan	2012	TARA_142_MES_0.22-3	640	Mesopelagic	25.63205	-88.42925	0.22-3	164,118,504	
	ERR868474	Jan	2012	TARA_142_MES_0.8-3	640	Mesopelagic	25.63205	-88.42925	> 0.8	179,903,731	

TABLE S2. Predicted taxonomy based on mapping to single-copy marker genes for archaeal and bacterial taxa classified at the phylum and class levels with >1% average relative abundance across the sample libraries. See **Data Set S1** for all results.

**Predicted Taxonomy**	**Average Relative Abundance (%) Across Libraries**	
**PHYLUM** Class	**Archaea**	**Bacteria**
**ACIDOBACTERIOTA**		**1**
**ACTINOMYCETOTA**		**5**
Acidimicrobiia		4
**BACTEROIDOTA**		**2**
Bacteroidia		2
**CHLOROFLEXOTA**		**3**
Dehalococcoidia		3
**MARINISOMATOTA (NOW FIDELIBACTEROTA)**		**8**
Marinisomatia		8
**NITROSPINOTA**		**2**
Nitrospinia		2
**PLANCTOMYCETOTA**		**3**
Planctomycetia		1
**PSEUDOMONADOTA**		**37**
Alphaproteobacteria		19
Gammaproteobacteria		18
**SAR324**		**5**
SAR324		5
**THERMOPLASMATOTA**	**5**	
Poseidoniia	5	
**THERMOPROTEOTA**	**21**	
Nitrososphaeria	21	
**VERRUCOMICROBIOTA**		**2**
Verrucomicrobiia		2
**UNASSIGNED**	**2**	

TABLE S3. Predicted taxonomy based on mapping to single-copy marker genes for bacterial and archaeal taxa classified at genus level with >1% average relative abundance across the libraries. See **Data Set S1** for all results.

**Predicted Taxonomy**	**Average Relative Abundance (%) Across Libraries**	
**PHYLUM,** Family, *Genus*	**Archaea**	**Bacteria**
**ACTINOMYCETOTA**		
MedAcidi-G1		
JAKUSA01		1
UBA9410		2
**CHLOROFLEXOTA**		
UBA3495		
UBA9611		1
**MARINISOMATOTA (NOW FIDELIBACTEROTA)**		
TCS55		
GCA-002701945		2
TCS55		2
D37C17		
D37C17		1
**PSEUDOMONADOTA**		
GCA-002718135		
AG-337-I02		1
MarineAlpha5-Bin3		1
Pelagibacteraceae		
AAA240-E13		1
AG-414-E02		6
Pelagibacter		5
UBA868		
CAJXWH01		1
REDSEA-S09-B13		1
Pseudothioglobaceae		
Pseudothioglobus		5
Pseudohongiellaceae		
UBA9145		1
SAR86		
AEGEAN-183		2
UBA11654		
DTSX01		1
**THERMOPLASMATOTA**		
CG-Epi1		
CG-Epi1	1	
Thalassarchaeaceae		
Thalassarchaeum	3	
**THERMOPROTEOTA**		
Nitrosopumilaceae		
Nitrosopelagicus	18	
Nitrosopumilus	1	
unassigned	18	

TABLE S4. Number of mapped reads and predicted taxonomy for archaeal and bacterial full-length 16S rRNA gene sequences assembled by phyloFlash for taxa with >10,000 mapped reads summed across the ten samples collected in this study. See **Data Set S2** for full results (398,318 reads total, including eukaryotic). Annotations are listed as returned by the analysis rather than updated nomenclatures.

**Assembled 16S rRNA gene sequences with >10,000 reads**	**Sum of mapped reads across libraries**	
**PHYLUM, Class,** ORDER, Family, *Genus*	**Archaea**	**Bacteria**
**ACTINOBACTERIOTA**		**14509**
**Acidimicrobiia**		14509
MICROTRICHALES		14509
Microtrichaceae		14509
Sva0996 marine group		14509
**CRENARCHAEOTA**	**69961**	
**Nitrososphaeria**	69961	
NITROSOPUMILALES	69269	
Nitrosopumilaceae	69269	
Thaumarchaeota archaeon SCGC AAA007-O23	24672	
uncultured archaeon	16821	
uncultured marine thaumarchaeote KM3_69_H10	10407	
**MARINIMICROBIA (SAR406 clade)**		**32796**
**uncultured bacterium**		17807
**PROTEOBACTERIA**		**164960**
**Alphaproteobacteria**		80569
RHODOSPIRILLALES		10001
SAR11 CLADE		64621
**Gammaproteobacteria**		84391
PSEUDOMONADALES		53706
SAR86 clade		11318
Thioglobaceae		33703
SUP05 cluster		33703
**SAR324 CLADE (MARINE GROUP B)**		**53866**
**THERMOPLASMATOTA**	**15728**	
**Thermoplasmata**	15728	
MARINE GROUP II	12181	
**Grand Total = 373,950**	**85689**	**288261**

TABLE S5. Metagenome-assembled genomes (MAGs) produced from the co-assembled metagenome of the ten samples collected in this study. See **Data Set S3** to expand results.

**Co-assembled MAGs**<br>**PHYLUM, Class,** ORDER, Family	**Sum of mapped reads across libraries**<br>**Archaea**	 **Bacteria**
**ACIDOBACTERIOTA**		**2**
**UBA890**		1
**Vicinamibacteria**		1
VICINAMIBACTERALES		1
UBA823		1
**ACTINOMYCETOTA**		**8**
**Acidimicrobiia**		8
ACIDIMICROBIALES		8
MedAcidi-G1		5
Poriferisodaliceae		3
**BACTEROIDOTA**		**1**
**Bacteroidia**		1
FLAVOBACTERIALES		1
UBA7430		1
**CHLOROFLEXOTA**		**5**
**Dehalococcoidia**		5
SAR202		1
UBA826		1
UBA1151		1
UBA3495		3
**MARINISOMATOTA (NOW FIDELIBACTEROTA)**		**9**
**Marinisomatia**		9
MARINISOMATALES		9
S15-B10		1
TCS55		3
UBA1611		4
UBA8229		1
**MYXOCOCCOTA_A**		**2**
**UBA9160**		**2**
**NITROSPINOTA**		**1**
**Nitrospinia**		1
NITROSPINALES		1
VA-1		1
**PLANCTOMYCETOTA**		**3**
**Planctomycetia**		2
PIRELLULALES		2
Pirellulaceae		1
UBA1268		1
**UBA8108**		**1**
**PORIBACTERIA**		**1**
**WGA-4E**		1
**PSEUDOMONADOTA**		**24**
**Alphaproteobacteria**		4
GCA-2731375		1
MICAVIBRIONALES		1
Micavibrionaceae		1
RHODOBACTERALES		1
Rhodobacteraceae		1

Co-assembled MAGs		Sum of mapped reads across libraries
RHODOSPIRILLALES_A		1
**Gammaproteobacteria**		**20**
ENTEROBACTERALES		1
Alteromonadaceae		1
METHYLOCOCCALES		1
PS1		3
Thioglobaceae		3
PSEUDOMONADALES		10
Azotimanducaceae_A		4
Nitrincolaceae		1
Oleiphilaceae		2
Porticoccaceae		2
Pseudohongiellaceae		1
SAR86		3
UBA11654		1
UBA4486		1
**SAR324**		**3**
**THERMOPLASMATOTA**	**9**	
**Poseidoniia**	9	
MGIII	1	
CG-Epi1	1	
POSEIDONIALES	8	
Poseidoniaceae	3	
Thalassarchaeaceae	5	
**THERMOPROTEOTA**	**2**	
**Nitrososphaeria**	2	
NITROSOSPHAERALES	2	
Nitrosopumilaceae	2	
**VERRUCOMICROBIOTA**		**1**
**Verrucomicrobiae**		1
VERRUCOMICROBIALES		1
Akkermansiaceae		1
**Grand Total**	**11**	**60**

TABLE S6. Linkages between taxonomic and functional annotations for the categories halogenated compound utilization, hydrocarbon degradation, and methane metabolism (**Data Set S4**). All annotations to these categories were classified as Gammaproteobacteria under phylum Pseudomonadota.

**Taxa**	**Halogenated**	**Hydrocarbon degradation**			**Methane Oxidation**
**CLASS, Order,** Family	**compound utilization**	**Alkane degradation**	**Alkene reduction**	**Cyclic alkane degradation**	
**GAMMAPROTEOBACTERIA**	**2**	**2**	**2**	**2**	**5**
**Methylococcales**					
UBA1147					3
**Pseudomonadales**					
Azotimanducaceae_A				2	2
Oleiphilaceae	1	1	1		
Porticoccaceae	1	1	1		

TABLE S7. Linkages between taxonomic and functional annotations for the category C1 metabolism classified as phylum Pseudomonadota (**Data Set S4**). See **Table S8** for other phyla.

Taxa	C1 metabolism		
**CLASS, Order, Family**	**Formaldehyde oxidation**	**Formate oxidation**	**Methanol oxidation**
**ALPHAPROTEOBACTERIA**	**6**	**5**	**1**
**GCA-2731375**	1	3	
GCA-2731375	1	3	
**Micavibrionales**	2		
Micavibrionaceae	2		
**Rhodobacterales**	2	2	1
Rhodobacteraceae	2	2	1
**Rhodospirillales_A**	1		
UBA2165	1		
**GAMMAPROTEOBACTERIA**	**18**	**12**	**11**
**Enterobacterales**	1		2
Alteromonadaceae	1		2
**Methylococcales**	4	2	2
UBA1147	4	2	2
**PS1**	2	2	
Thioglobaceae	2	2	
**Pseudomonadales**	10	7	5
Azotimanducaceae_A	4	2	2
Nitrincolaceae	1	1	1
Oleiphilaceae	2	2	
Porticoccaceae	2	1	1
Pseudohongiellaceae	1	1	1
**SAR86**	1		1
SAR86	1		1
**UBA11654**		1	
UBA11654		1	
**UBA4486**			1
UBA4486			1

TABLE S8. Linkages between taxonomic and functional annotations for the category C1 metabolism classified to phyla other than those shown in **Table S7** (Pseudomonadota).

Taxa<br>**PHYLUM, Class,** Order, Family	C1 metabolism<br>Formaldehyde oxidation	 Formate oxidation	 Methanol oxidation
**ACIDOBACTERIOTA**			**1**
**UBA890**			1
UBA890			1
UBA890			1
**ACTINOMYCETOTA**	**6**		
**Acidimicrobiia**	**6**		
Acidimicrobiales	6		
MedAcidi-G1	3		
Poriferisodaliceae	3		
**CHLOROFLEXOTA**	**4**	**3**	
**Dehalococcoidia**	**4**	**3**	
SAR202		1	
UBA826		1	
UBA1151	1		
UBA1328	1		
UBA3495	3	2	
UBA3495	3	2	
**MYXOCOCCOTA_A**	**2**		**1**
**UBA9160**	**2**		**1**
UBA9160	2		1
UBA4427	2		1
**NITROSPINOTA**		**1**	
**Nitrospinia**		**1**	
Nitrospinales		1	
VA-1		1	
**PLANCTOMYCETOTA**	**4**		
**Planctomycetia**	**3**		
Pirellulales	3		
Pirellulaceae	2		
UBA1268	1		
**UBA8108**	**1**		
UBA8108	1		
UBA8108	1		
**PORIBACTERIA**			**1**
**SAR324**	**3**	**4**	
**SAR324**	**3**	**4**	
SAR324	3	4	
NAC60-12	3	4	
**THERMOPLASMATOTA**		**3**	
**Poseidoniia**		**3**	
Poseidoniales		3	
Poseidoniaceae		1	
Thalassarchaeaceae		2	

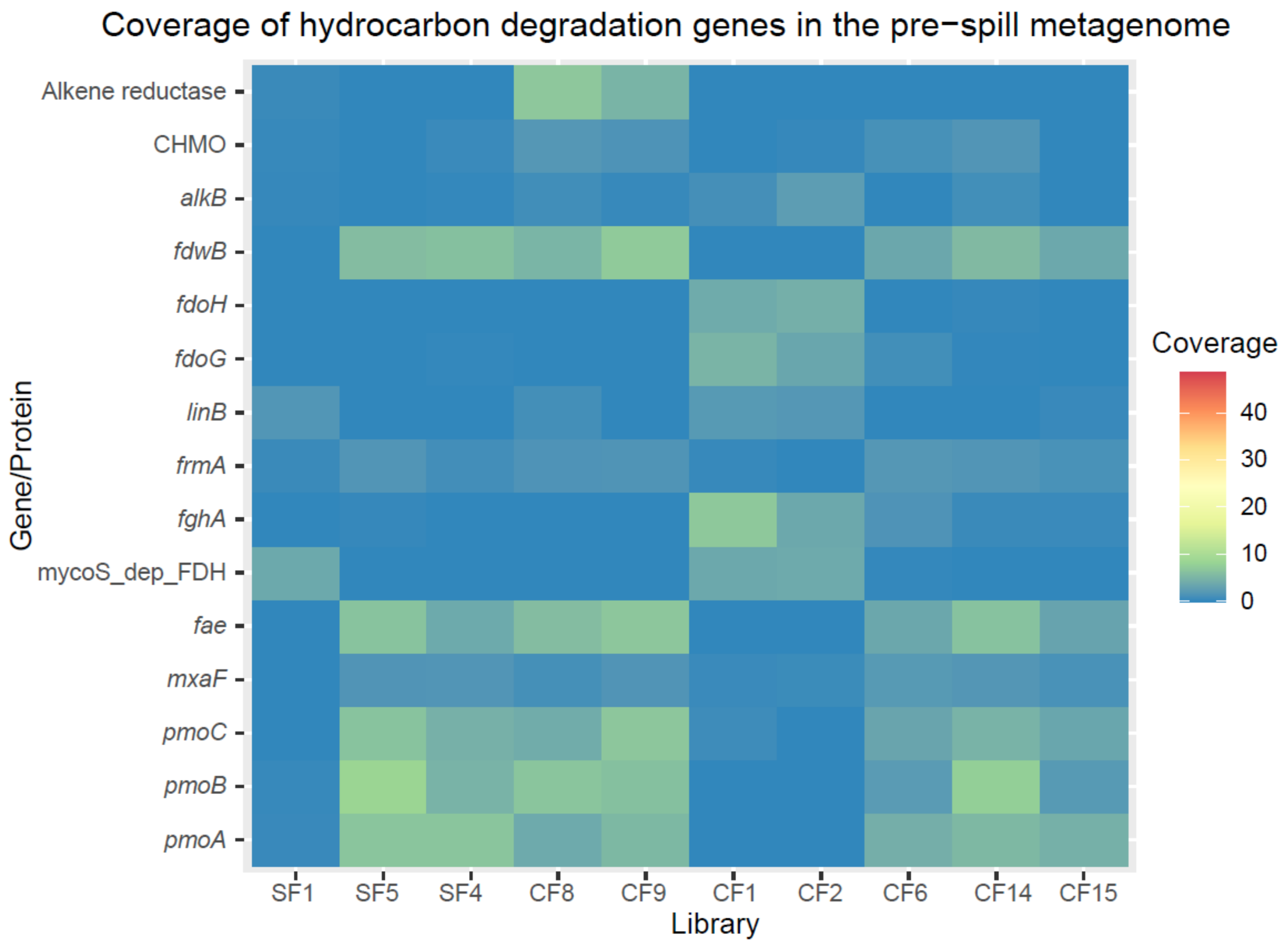

FIGURE S1 Mean coverage (average depth of coverage across contig) values of genes involved in C1-compound oxidation and alkane degradation in MAGs assembled in this study. Abbreviations are listed in **Data Set S4**. Samples were collected from the Green Canyon lease area water column >600 m depth (CF6, CF14, CF15), including above (SF4, SF5) or beside (CF8, CF9) Brine Pool NR-1. SF1, CF1, and CF2 were collected at more shallow depths (<200 m), with SF1 collected from a CTD cast deployed above Brine Pool NR-1 (**Table 1** and **Table S1**).

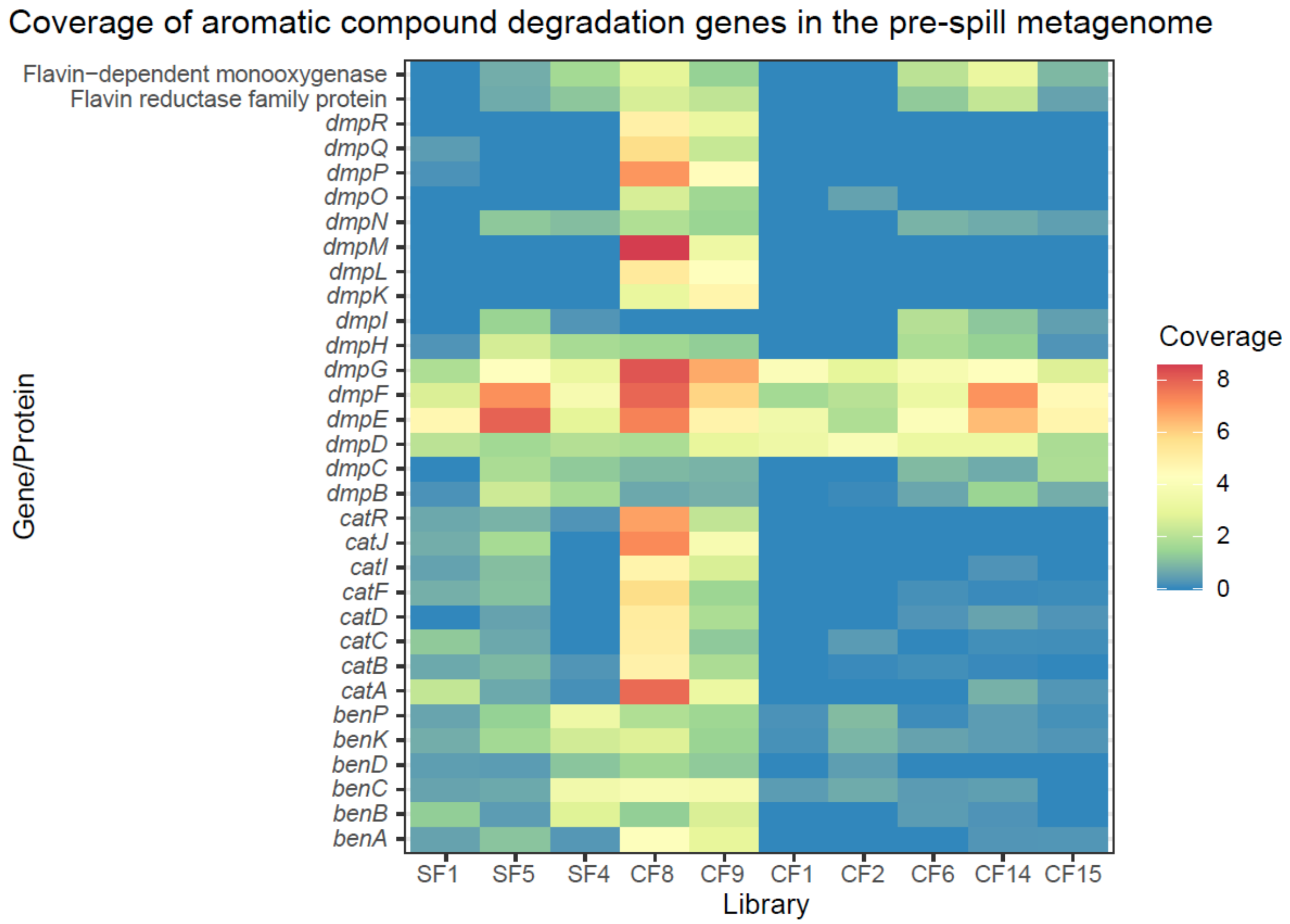

FIGURE S2 Mean coverage (average depth of coverage across contig) values of genes involved in aromatic compound degradation in MAGs assembled in this study. Abbreviations are listed in **Data Set S4**. Samples were collected from the Green Canyon lease area water column >600 m depth (CF6, CF14, CF15), including above (SF4, SF5) or beside (CF8, CF9) Brine Pool NR-1. SF1, CF1, and CF2 were collected at more shallow depths (<200 m), with SF1 collected from a CTD cast deployed above Brine Pool NR-1 (**Table 1** and **Table S1**).

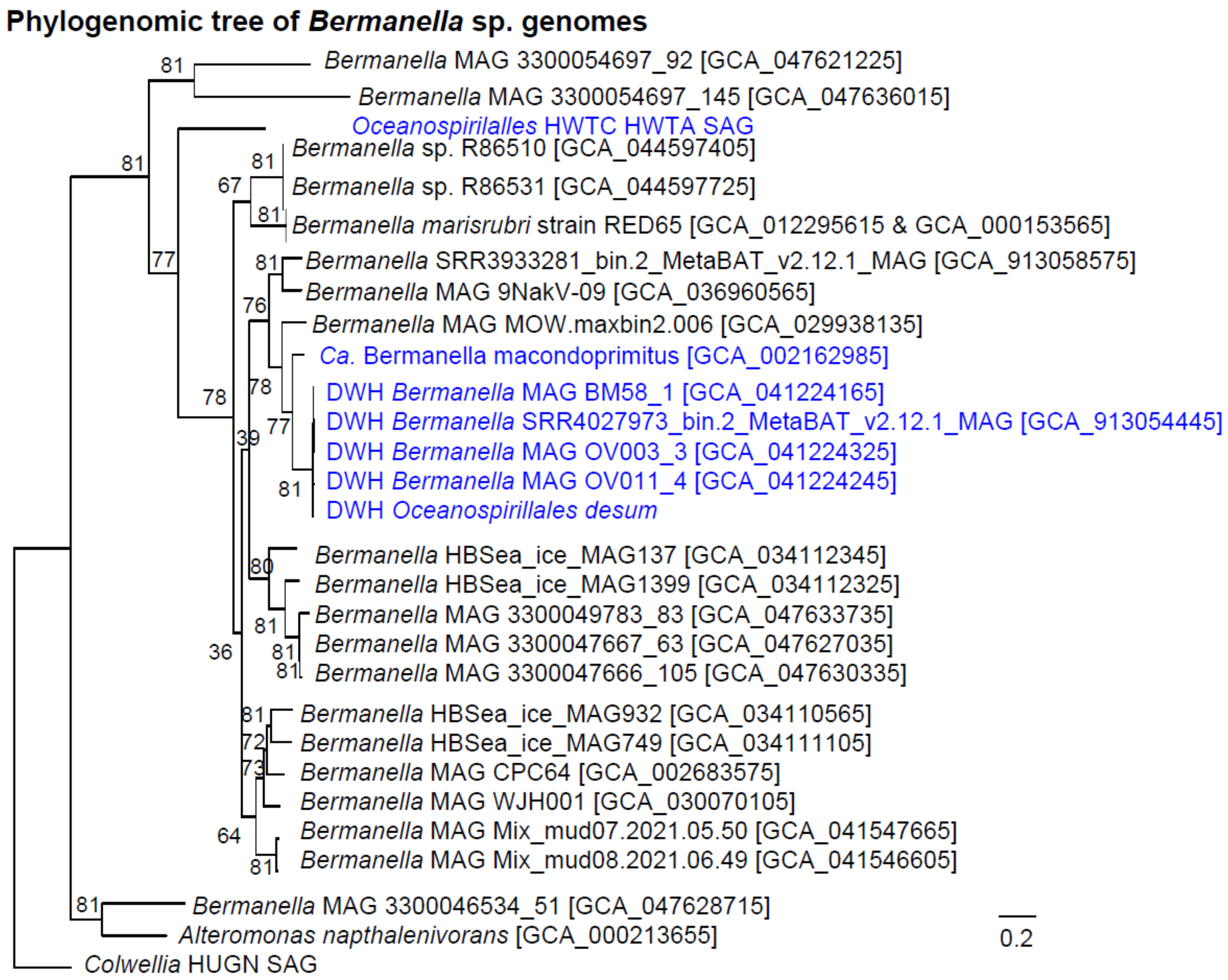

FIGURE S3 Phylogenomic tree of publicly available *Bermanella* sp. genomes, in relation to genome relatives assembled from plume samples collected during the spill. The genome of *Alteromonas napthalenivorans* (order: Alteromonadales) and the SAG of *Colwellia*

sp. (order: Enterobacterales) recovered from plume samples collected during the spill were used as the outgroup. NCBI accession numbers, if available, for genomes listed on the tree are indicated in square brackets. *Bermanella* sp. genomes highlighted in blue were assembled from plume samples during the spill or from indigenous seawater collected from the spill site at plume depth and incubated with microdroplets of Macondo oil and Corexit for 6 days (*Ca.* Bermanella macondoprimitus). The tree was generated from the concatenated codon-based sequence alignment (69,777 bp) of 81 marker genes detected by UBCG2. Node values indicate the Gene Support Index (GSI), which represents the number of gene trees (maximum 81) supporting each branch in the final tree. The scale bar indicates the number of substitutions per site.

Description of Supplementary Data Sets

Data Set S1

Domain: Relative abundance and taxonomy of each OTU, collapsed at the domain level, predicted by SingleM from marker-specific OTU tables for all 10 libraries analyzed in this study.
Phylum: Relative abundance and taxonomy of each OTU, collapsed at the phylum level, predicted by SingleM from marker-specific OTU tables for all 10 libraries analyzed in this study.
Class: Relative abundance and taxonomy of each OTU, collapsed at the class level, predicted by SingleM from marker-specific OTU tables for all 10 libraries analyzed in this study.
Order: Relative abundance and taxonomy of each OTU, collapsed at the order level, predicted by SingleM from marker-specific OTU tables for all 10 libraries analyzed in this study.
Family: Relative abundance and taxonomy of each OTU, collapsed at the family level, predicted by SingleM from marker-specific OTU tables for all 10 libraries analyzed in this study.
Genus: Relative abundance and taxonomy of each OTU, collapsed at the genus level, predicted by SingleM from marker-specific OTU tables for all 10 libraries analyzed in this study.

Data Set S2

phyloFlash_SSUfull_length: Number of mapped reads, coverage, closest sequence relative, predicted taxonomy, and BLAST results (% identity, alignment length, and expect value) of full-length 16S rRNA gene sequences generated by phyloFlash assembly of reads sequenced from the ten samples collected in this study.
phyloFlash_summary: Total number of mapped reads for each reference sequence matching the full-length 16S rRNA gene sequences generated by phyloFlash assembly of reads sequenced from the ten samples collected in this study. The total number of mapped reads is calculated by adding up the number of mapped reads for each sequence relative across all samples analyzed.

Data Set S3

QUAST: Quality of the metagenome of the 10 samples collected in this study prior to the Deepwater Horizon oil spill and the 12 post-spill samples collected at Tara station 142 co-assembled using MEGAHIT and assessed using QUAST.
Prespill MAGs: Metagenome-assembled genomes (MAGs) produced from the co-assembled metagenome of the 10 samples collected in this study using MEGAHIT and binned using MetaBat2, MaxBin2, DAS Tool. MAG quality was evaluated using anvi'o and checkM, and MAG taxonomy was assigned using GTDB-TK based on GTDB release 220.
***Bermanella* recruitment:** The percentage of reads from each of the 65 metagenomic libraries (rows) that mapped to contigs in each of the *Bermanella* MAGs analyzed (columns), out of all mapped reads for that specific library.

Data Set S4

Hydrocarbon: Functional annotations and mean coverage (average depth of coverage across contig; last 10 columns) values of genes involved in C1-compound oxidation and alkane degradation in MAGs assembled from the metagenome generated from the ten samples collected in this study. Functional annotations were generated by anvi'o, METABOLIC, and PGAP, and verified by web blast searches.

Aromatic: Functional annotations and mean coverage (average depth of coverage across contig; last 10 columns) values of aromatic compound degradation genes in MAGs assembled from the metagenome generated in this study. Functional annotations were generated by anvi'o, METABOLIC, and PGAP, and verified by web blast searches.

References

1. Eren AM, Esen OC, Quince C, Vineis JH, Morrison HG, Sogin ML, Delmont TO. 2015. Anvi'o: an advanced analysis and visualization platform for 'omics data. PeerJ 3:e1319.
2. Shapiro SS, Wilk MB. 1965. An analysis of variance test for normality (complete samples). Biometrika 52:591-611.
3. Mann HB, Whitney DR. 1947. On a test of whether one of two random variables is stochastically larger than the other. Annals of Mathematical Statistics 18:50-60.
4. Beghini F, McIver LJ, Blanco-Míguez A, Dubois L, Asnicar F, Maharjan S, Mailyan A, Manghi P, Scholz M, Thomas AM, Valles-Colomer M, Weingart G, Zhang Y, Zolfo M, Huttenhower C, Franzosa EA, Segata N. 2021. Integrating taxonomic, functional, and strain-level profiling of diverse microbial communities with bioBakery 3. eLife 10:e65088.
5. Caspi R, Billington R, Keseler IM, Kothari A, Krummenacker M, Midford PE, Ong WK, Paley S, Subhraveti P, Karp PD. 2020. The MetaCyc database of metabolic pathways and enzymes - a 2019 update. Nucleic Acids Res 48:D445-D453.
6. Chaumeil PA, Mussig AJ, Hugenholtz P, Parks DH. 2019. GTDB-Tk: a toolkit to classify genomes with the Genome Taxonomy Database. Bioinformatics doi:10.1093/bioinformatics/btz848.
